# Hybrid Graphene-Supported Aluminum Plasmonics

**DOI:** 10.1021/acsnano.2c01730

**Published:** 2022-07-29

**Authors:** Kenan Elibol, Peter A. van Aken

**Affiliations:** Stuttgart Center for Electron Microscopy, Max Planck Institute for Solid State Research, Heisenbergstr. 1, 70569 Stuttgart, Germany

**Keywords:** localized surface plasmon resonance, electron energy-loss spectroscopy, nanofabrication, graphene, monochromated scanning transmission electron microscopy

## Abstract

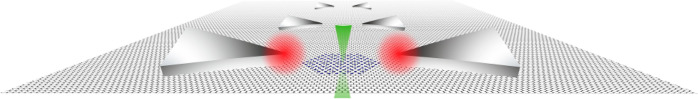

Controlled fabrication of devices for plasmonics on suspended graphene enables obtaining tunable localized surface plasmon resonances (LSPRs), reducing the red-shift of LSPRs, and creating hybrid 3D–2D systems promising for adjustable dipole–dipole coupling and plasmon-mediated catalysis. Here, we apply a low-cost fabrication methodology to produce patterned aluminum nanostructures (bowties and tetramers) on graphene monolayers via electron-beam lithography and trap platinum (Pt) nanoclusters (NCs) within their hotspots by thermal annealing. We reveal the LSPRs of aluminum plasmonics on graphene using electron energy-loss spectroscopy (EELS) and energy-filtered transmission electron microscopy (EFTEM) in a monochromated scanning transmission electron microscope (STEM). The LSPRs of these nanostructures are measured to be between visible and ultraviolet regions of the spectrum and are confirmed by electromagnetic simulations. The antibonding dipole and bonding dipole modes of both structures are tuned by controlling their gap size. The tetramers enable the simultaneous excitation of both antibonding and bonding dipole modes at the poles of nanoprisms, while bowties allow us to excite these modes separately either at the poles or within the hotspot. We further show that the hybrid nanocavity–NC systems are in the intermediate coupling regime providing an enhanced plasmon absorption in the Pt NCs via the energy transfer from the antibonding dipole mode to the Pt NCs. The dipole LSPR of Pt NCs also couples to the bonding-type breathing mode in bowties. Our findings suggest that these hybrid nanocavity–graphene systems are of high application potential for plasmon-mediated catalysis, surface-enhanced fluorescence, and quantum technologies.

## Introduction

The strength of light–matter interactions can be enhanced via plasmonic nanocavities created by closely spaced metal nanoparticles enabling excitation of localized surface plasmon resonances (LSPRs).^1−3^ These nanocavities are often used to explore various phenomena such as cavity quantum electrodynamics, strong coupling, and ultrafast surface-enhanced fluorescence.^2,4−6^ Strong coupling of trapped species, e.g., atoms, molecules, and nanoclusters (NCs), in plasmonic nanocavities can also support the construction of quantum networks to probe fundamentals of quantum communication and quantum information processing.^7−10^ The bonding dipole modes can be excited by a plane wave, whereas the antibonding dipole modes that behave like an energy sink for photon emitters in close proximity cannot couple to the far-field.^11−13^ The antibonding dipole modes with extremely high-quality factors and longer lifetimes can thus find applications in various fields such as single-emitter strong coupling, energy transport, biosensing, and nonlinear plasmonics.^14−17^ Additionally, they enable the efficient absorption of photons as well as the generation of hot electrons and localized heat.^18,19^

Contrary to gold (Au) and silver (Ag), which are often used as a material for plasmonics, aluminum (Al) is a low-cost and earth-abundant material that can support ultraviolet (UV) and visible plasmon excitations.^20^ The LSPRs excited in plasmonics depend strongly on the underlying substrate and its thickness, which leads to a red-shift of the LSPRs and a mode mixing resulting from the coupling of a plasmon mode with its own image charge and other plasmon modes.^21−24^ The most common electron-transparent substrate used for examining plasmonics in transmission electron microscopy (TEM) is SiN_*x*_ with a dielectric constant of ∼4.25,^25,26^ which can be produced up to a minimum thickness of 3 nm.^27^ However, it is thicker than two-dimensional (2D) materials, especially one-atom-thick graphene and hexagonal boron nitride (h-BN).^28,29^ Graphene is known as the strongest material,^30^ and it is more stable than h-BN under electron-beam irradiation.^31^ Therefore, the imaging of substitutional impurity atoms and atomic clusters on graphene is possible by TEM.^32,33^ Moreover, it is a great substrate for electron-beam-patterned nanostructures.^34^ Combining the LSPRs and superior electrical and optical properties of 2D materials such as graphene enables stronger confinement and manipulation of electric fields.^35^ Even though graphene is a promising substrate for plasmonics, the large-area fabrication of patterned nanostructures on atomically clean chemical vapor deposition (CVD)-grown monolayer graphene membranes has not been achieved yet.

STEM is one of the most versatile tools to probe individual atoms as well as molecules/metal clusters and to monitor their dynamics at the atomic scale.^32,33,36,37^ It also enables mapping the chemical composition of a specimen with single-atom sensitivity by EELS and mapping the excitation of antibonding dipole modes, which cannot be directly excited by light.^38,39^ Recent studies show that the strong coupling of excitons in bulk semiconductors, e.g., ZnO, or 2D materials, e.g., WS_2_, to LSPRs of Ag nanoparticles can be examined by EELS in a monochromated and aberration-corrected STEM.^40,41^ Strong coupling also occurs between LSPR modes, e.g., dipole–dipole coupling in Au nanorods and dipole–anapole coupling in the Au nanorods–Si nanodisks system, via plasmonic interactions inducing a Rabi splitting.^12,42^ Though strong coupling has been achieved by placing organic dye molecules, semiconductor quantum dots (QDs), and 2D materials nearby plasmonic nanoparticles,^41,43−45^ the strong coupling between excitonic states or dipolar modes of metal NCs and dipolar LSPRs of plasmonic nanocavities has remained elusive to date. The hybrid plasmonics with 2D materials are promising not only for strong coupling but also for enhanced spectroscopy.^46−49^

When the size of metal clusters is reduced to 2 nm or below, they present molecule-like characteristics such as single-electron transitions and excitons due to discrete energy levels in their electronic structure, and they drastically lose their plasmonic nature in the quantum-size regime.^50^ Smaller metal NCs also show higher catalytic activity due to their high surface-to-volume ratio.^51^ Pt NCs are often used as a catalyst for photocatalytic and electrocatalytic reactions.^52−54^ The catalytic activity of Pt NCs becomes higher when their LSPRs and/or hot electrons are activated via a plasmonic nanocavity in close proximity.^51,55^ The LSPR and/or hot-electron-mediated activation of the catalytic reactions also enables creating nanozymes.^55^

Here, we present a low-cost fabrication methodology to produce large-area plasmonic arrays consisting of Al bowties and tetramers on atomically clean graphene monolayers attached to a TEM grid. The hydrocarbon adsorbates on graphene are removed via the activation of a catalytic Pt layer on the TEM grid at 300 °C for 15 min. Thermally activated Pt NCs diffusing on graphene are agglomerated around the hotspots of nanocavities due to the high surface reactivity of Al_2_O_3_ forming on metallic Al. The LSPRs of Al plasmonics on graphene are visualized by EELS and EFTEM measurements in a monochromated STEM/TEM and are supported and analyzed via boundary element method (BEM) simulations. BEM simulations of EEL spectra ascertain that monolayer graphene underlying Al nanocavities induces less red-shift in LSPRs compared to monolayer h-BN and 3 nm thick SiN_*x*_. The antibonding dipole LSPRs of both bowties and tetramers on graphene can be tuned by controlling the gap size, while the energy of the bonding dipole mode in these structures is nearly independent of the gap size. The lifetime and quality factor (*Q*) of antibonding dipole modes in both nanocavities are higher than those of bonding dipole modes, and they increase when the gap size is reduced. Contrary to bowties, the tetramers with lower *Q* enable producing dipolar modes in a wide energy range of 1.84–2.65 eV as well as the simultaneous excitation of both antibonding and bonding dipole modes at the poles of nanoprisms. With the coupling of dipole LSPRs of Pt NCs to the antibonding dipole modes of bowties, we reveal intermediate dipole–dipole coupling in the nanocavity–NC systems. Moreover, we observe the coupling of a bonding breathing mode in a bowtie to a dipolar mode in a Pt NC. The proposed hybrid nanocavity–graphene systems allow (i) revealing dynamic catalytic processes of single atoms and molecules confined within the plasmonic hotspots, (ii) revealing weak, intermediate, and strong coupling phenomena, and (iii) controlling the enzyme-like catalytic activity of trapped species.

## Results and Discussion

### Large-Area Fabrication of Al Plasmonics on CVD Graphene

**Figure 1:**
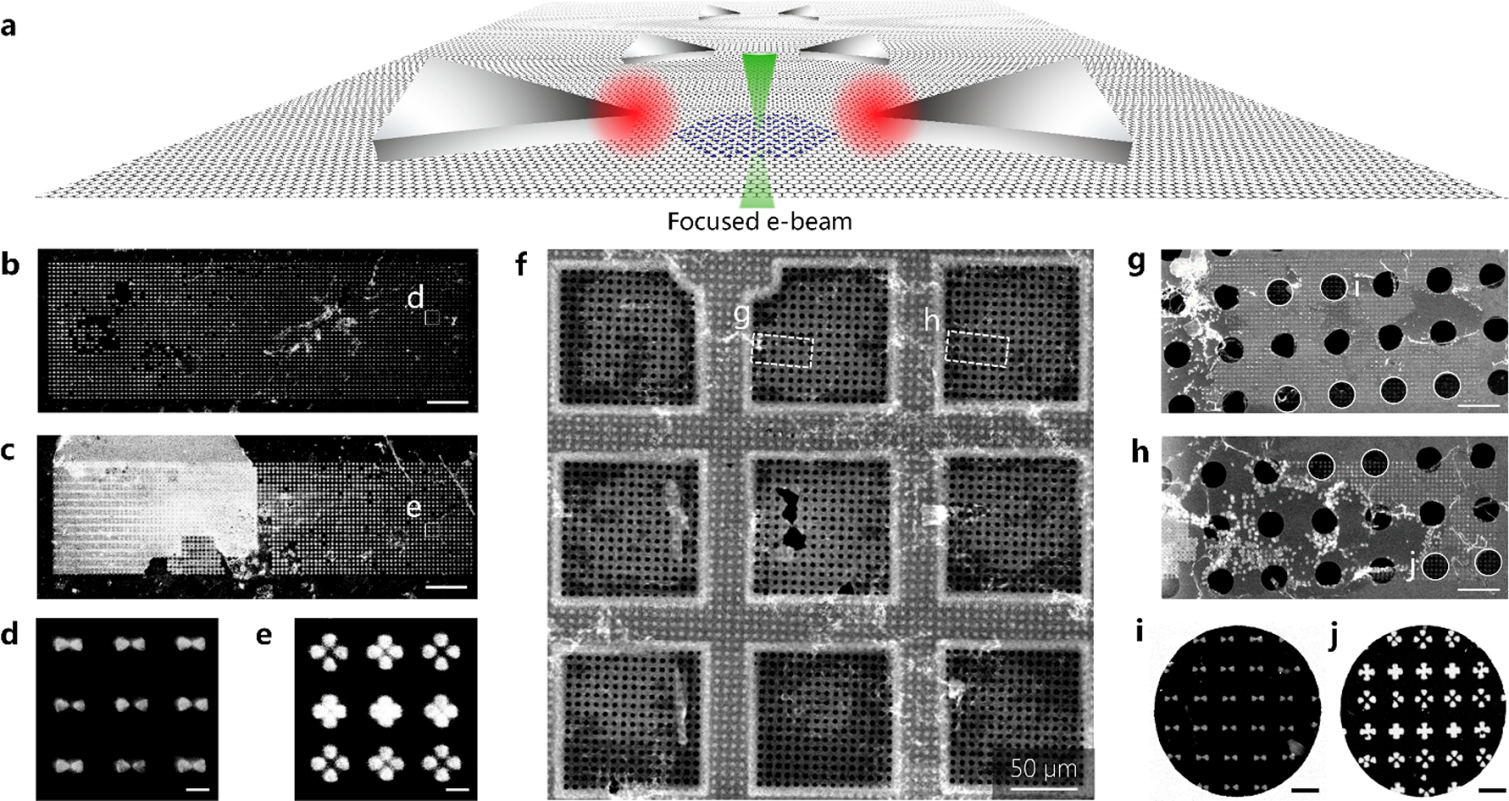
(a) Schematic of the Al bowtie nanocavities coupled to Pt NCs on a monolayer graphene membrane. (b, c) SEM images of bowtie and tetramer arrays fabricated on CVD-grown graphene on a Cu foil. The scale bars are 5 μm. (d, e) Close-up SEM images recorded at areas marked by white squares in panels b and c. The scale bars are 100 nm. (f) SEM image of the same nanocavity arrays transferred on a Au TEM grid with a perforated support film. (g, h) SEM images of bowtie and tetramer arrays marked by white dashed rectangles in panel f. The scale bars are 5 μm. The areas covered by graphene are marked by white circles. (i, j) Close-up HAADF-STEM images of Al bowtie and tetramer nanocavities recorded at areas marked on panels g and h. The scale bars are 500 nm.

Figure 1a shows the schematic of the experiment carried out. Here, the Al plasmonics bearing nanocavities are fabricated on suspended monolayer graphene by electron-beam lithography. Pt NCs are trapped within the plasmonic hotspots to reveal the weak and strong coupling by low-loss EELS measurements (for detailed information, see the Methods section and Figure S1). The gap size of these nanocavities is controlled by varying the beam dose during the patterning of the PMMA (see the Methods section). As shown in Figure 1b,c (see also Figure S2), the largest gaps are obtained in the areas that are patterned at low beam doses of <1400 μC/cm^2^, while there is no gap for patterned nanostructures at beam doses of >1704 μC/cm^2^ (for tetramers) and >2820 μC/cm^2^ (for bowties). The long bright lines observed in the SEM images (see Figure 1c and Figure S2) are grain boundaries of the Cu foil. In Figure 1c, the white area partially covering the tetramer nanocavity array is an Al film, which cannot be removed during the lift-off process (see also Figure S3). To probe the plasmon modes of Al plasmonics, we transfer the nanocavity/graphene stack onto a Au TEM grid with a perforated support film including 3 μm holes (Quantifoil). Figure 1f–h shows the SEM images of the nanocavity arrays transferred onto the TEM grid. The hole observed at the center of the grid (see the black area on panel f) is created by the needle of a home-designed micromanipulator used for positioning the TEM grid with respect to the plasmonic arrays on graphene/Cu. Close-up HAADF-STEM images of both bowtie and tetramer nanocavities are shown in Figure 1i,j. Detailed HAADF-STEM images of nanocavity arrays transferred on a TEM grid are presented in Figure S4. Although most of the nanocavities remain stable after the transfer, some areas on the holey TEM support film have nanocavities distributed randomly (Figure S4d). This is due to mechanical forces, which can create local distortions and cracks in graphene during the transfer process (Figure S5a). These local deformations, however, do not affect the majority of nanocavities (Figure S5a,b).

### Trapping of Pt NCs within Plasmonic Hotspots

**Figure 2:**
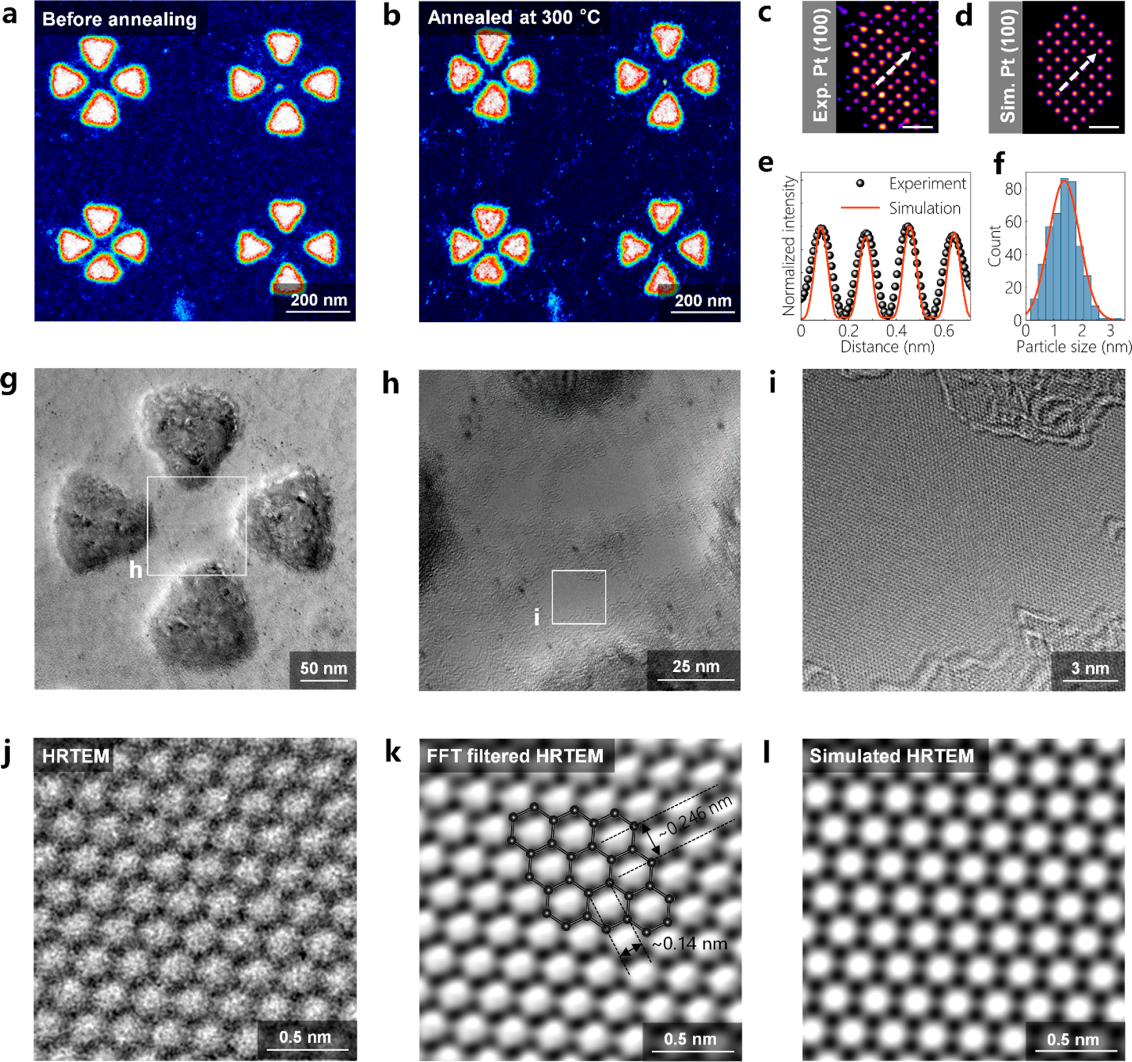
(a, b) TEM image of Al tetramer nanocavities on graphene before and after thermal annealing at 300 °C for 15 min, respectively. (c) Filtered HAADF-STEM image of a Pt NC formed on graphene after thermal annealing. The image is filtered by double Gaussian filtering (σ1 = 0.26, σ2 = 0.20, weight = 0.10) and a Wiener filtering to reduce the noise and to enhance the image contrast. (d) Simulated HAADF image of a Pt(100) NC. The scale bars are 0.5 nm. (e) Line profiles recorded along the white dashed arrows on the experimental and simulated HAADF images. (f) Histogram showing the size distribution of Pt NCs on graphene. (g) TEM image of an Al tetramer nanocavity on graphene. (h, i) HRTEM images acquired at the plasmonic hotspot of the tetramer. (j, k) Raw and FFT-filtered HRTEM images of the monolayer graphene at the plasmonic hotspot. An atomistic model of graphene is superimposed on the TEM image in panel k. (l) Simulated HRTEM image of the monolayer graphene.

To remove hydrocarbon contamination and polymer residues on the graphene membrane, the sample (nanocavity/graphene attached to the TEM grid) is annealed at 300 °C in the air for 15 min. Here, a 10 nm thick Pt layer covered on the TEM support film (see the Methods section and Figure S1 for details) is used as a catalyst to dissociate molecular-adsorbed H_2_ into atomic hydrogen.^56^ As shown in Figure 2a,b, thermal annealing enables obtaining large clean areas on the graphene membrane. However, heating at ambient conditions leads to the formation of cracks on the TEM support film (Figure S6a,b). Note that the support film remains undistorted when the TEM grid is heated in a vacuum. The small bright spots observed on graphene (Figure 2b) are Pt NCs formed after annealing. An atomic-resolution HAADF image of a Pt NC is shown in Figure 2c. The simulated HAADF image confirms that the Pt NC is oriented along the ⟨100⟩-direction (Figure 2d,e). The Pt NCs observed on graphene are either ⟨100⟩- or ⟨110⟩-oriented (Figure S7a–d). The existence of Pt is further confirmed via EDS measurements (Figure S7e). The heating process also enables the trapping of individual Pt NCs within the plasmonic hotspots of e-beam-fabricated Al nanocavities (Figure S8). The average size of Pt NCs with planar hexagonal shapes on graphene (see also Figure S7b) is measured to be 1.33 ± 0.04 nm (Figure 2f). Figure 2g shows the HRTEM image of a tetramer located on suspended monolayer graphene. Here, the thickness of Al nanoprisms is about ∼30 nm as determined by EFTEM thickness mapping (Figure S9a–h), which is in good agreement with the nominal thickness of ∼30 nm observed on the quartz microbalance of the thermal evaporator. There is, however, a ∼10 nm thick amorphous aluminum oxide (AlO_*x*_) layer covering the surface of metallic aluminum (Figure S10). This oxide layer induces a red-shift in the LSPRs of the nanocavity, but it also acts as a protective layer for the metallic Al surface.^57,58^ Close-up HRTEM images of the plasmonic hotspot at one of the tetramer nanocavities are shown in Figures 2h,i. The small black dots appearing at the hotspot area are Pt NCs on hydrocarbon contamination at the graphene surface (Figure 2h). A contamination-free area within the hotspot (marked with a white square in Figure 2h) is shown in Figure 2i. The carbon atoms in graphene explicitly appear in the atomic-resolution HRTEM image (Figure 2j,k), which shows a good match with the simulated HRTEM image of monolayer graphene (Figure 2l).

### Visualizing Plasmon Modes at Al Nanocavities on Graphene

**Figure 3:**
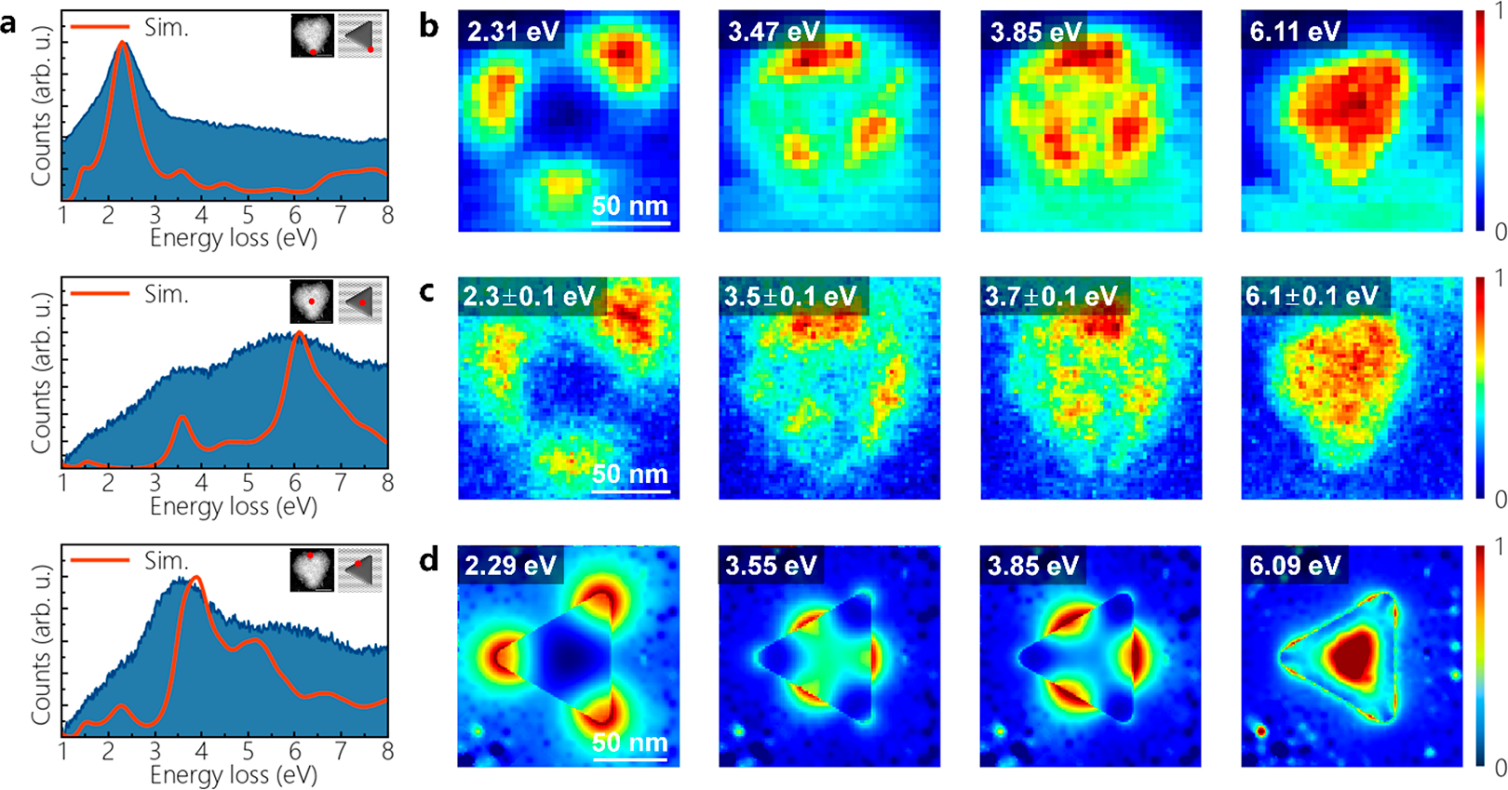
(a) EEL spectra acquired at three different positions at the Al nanoprism (see red dots in HAADF images). The scale bars in the HAADF images are 50 nm. The red curves are the simulated EEL spectra calculated at the same positions in the models, which are similar to the experimental structure (see insets). (b) Spatially resolved EELS maps acquired at different energies. (c) EFTEM maps acquired at different energies on the same sample. (d) Simulated EELS maps calculated at different loss energies. Experiments and simulations are performed at an accelerating voltage of 200 kV (see the Methods section for details).

The influence of the substrate on the LSPRs of an Al nanoprism placed on different membranes (3 nm thick SiN_*x*_, monolayer graphene, and monolayer h-BN) is inspected via BEM simulations of EEL spectra in Figure S11a–g. The simulations indicate that a 3 nm thick SiN_*x*_ induces the largest red-shift in LSPRs, whereas a monolayer graphene membrane leads to the least red-shift. Based on the BEM simulations, the monolayer graphene is found to be the best substrate for Al plasmonics. Figure 3a shows the low-loss EEL spectra acquired at different positions on the Al nanoprism. The red curves correspond to simulated EELS spectra. The Al nanoprism on graphene exhibits LSPRs at the energies of 2.31 ± 0.01, 3.47 ± 0.01, 3.85 ± 0.1, and 6.11 eV. Spatially resolved EELS maps acquired at these energies show that the LSPR observed at 2.31 eV corresponds to the dipolar mode, while edge LSPR modes are visible at energies of 3.47 and 3.85 eV (Figure 3b). The LSPR appearing at 6.12 eV is a breathing mode. Lastly, the peak at ∼1.46 eV is attributed to an interband excitation (IBT) of Al (Figure 3a). The EFTEM maps acquired at the same nanocavity support the LSPR modes detected via EELS mapping (Figure 2c). Here, all EELS measurements are performed using a monochromated STEM with an energy resolution of 0.17 eV (see Figure S12). Instead, EFTEM measurements are carried out with a maximum energy resolution of 0.23 eV. These LSPR modes are further confirmed via simulated EELS maps calculated at energies obtained from BEM simulations of EEL spectra shown in Figure 3a. We stress that the slight mismatch between experimental and simulated EEL spectra is due to the deviations in the dimensions of the experimental structure and the dielectric function of Al used in BEM simulations. The intensity variations on the graphene membrane (Figure 3d) might be due to the size effect of the graphene membrane, which is about 150 × 150 nm^2^ in simulations.

**Figure 4:**
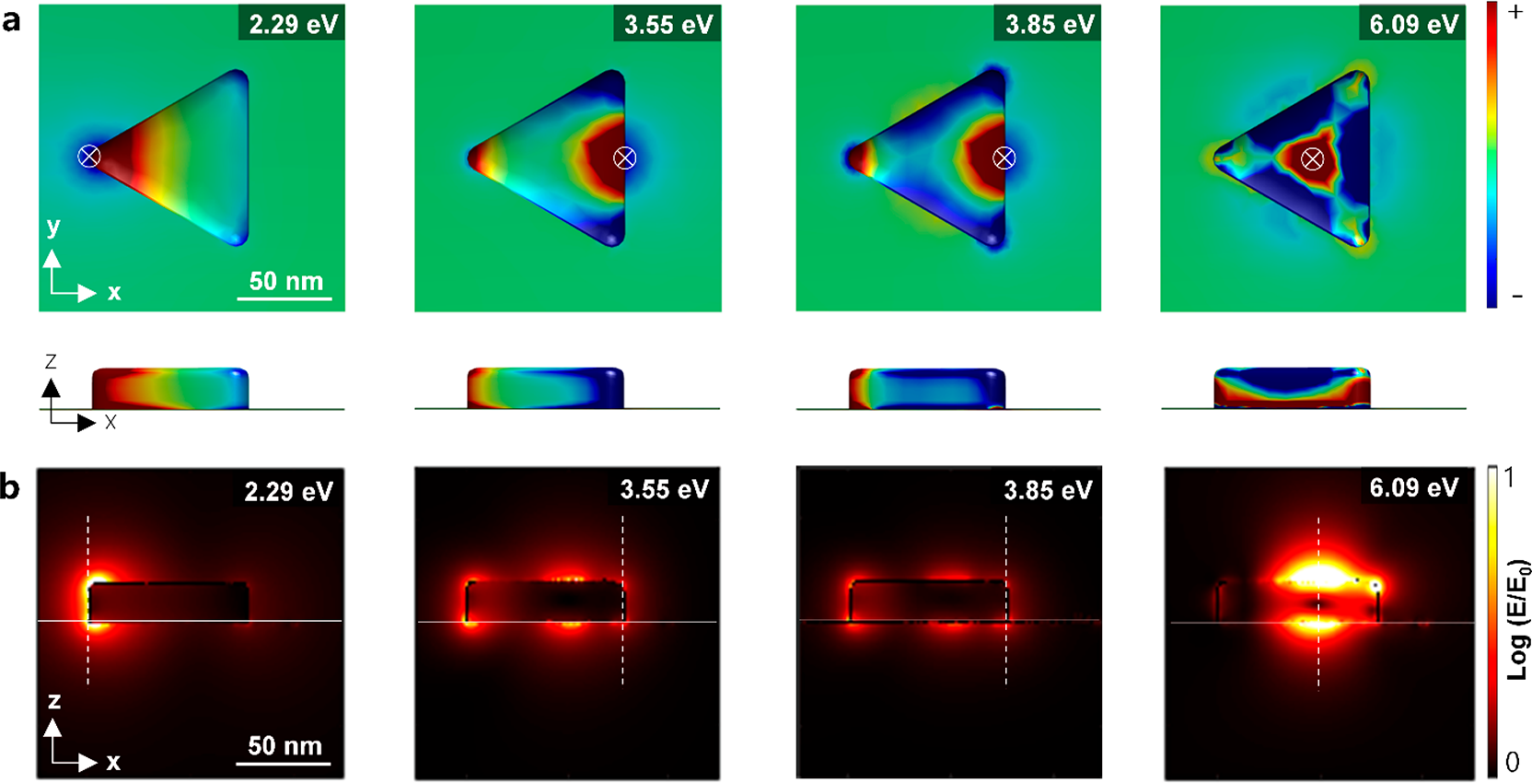
(a) Plane and side views of the simulated surface-charge distribution of an Al nanoprism on monolayer graphene excited at different energies. The direction of the electron beam is shown by the symbol ⊕. (b) Simulated electric field maps of the same structure excited at different energies. The electron trajectories in panel b are shown by vertical dashed white lines. The graphene membrane is represented with a lateral solid white line.

The simulated surface-charge distribution and electric field maps of a single Al nanoprism on graphene are shown in Figure 4. As shown in Figure 4a, the electron beam exiting the LSPRs of an Al nanoprism induces negative and positive charges on the nanoprism, but the graphene membrane remains mostly neutral. However, with the excitation of especially the high-energy breathing mode at 6.09 eV, some areas on graphene nearby the nanoprism become charged. The electric-field distribution shows that the graphene membrane underlying the Al nanoprism also leads to a slight decrease of the electric field in the −*z* direction (Figure 4b). The reduction of the induced electric field is most pronounced in the field map calculated at 6.09 eV.

**Figure 5:**
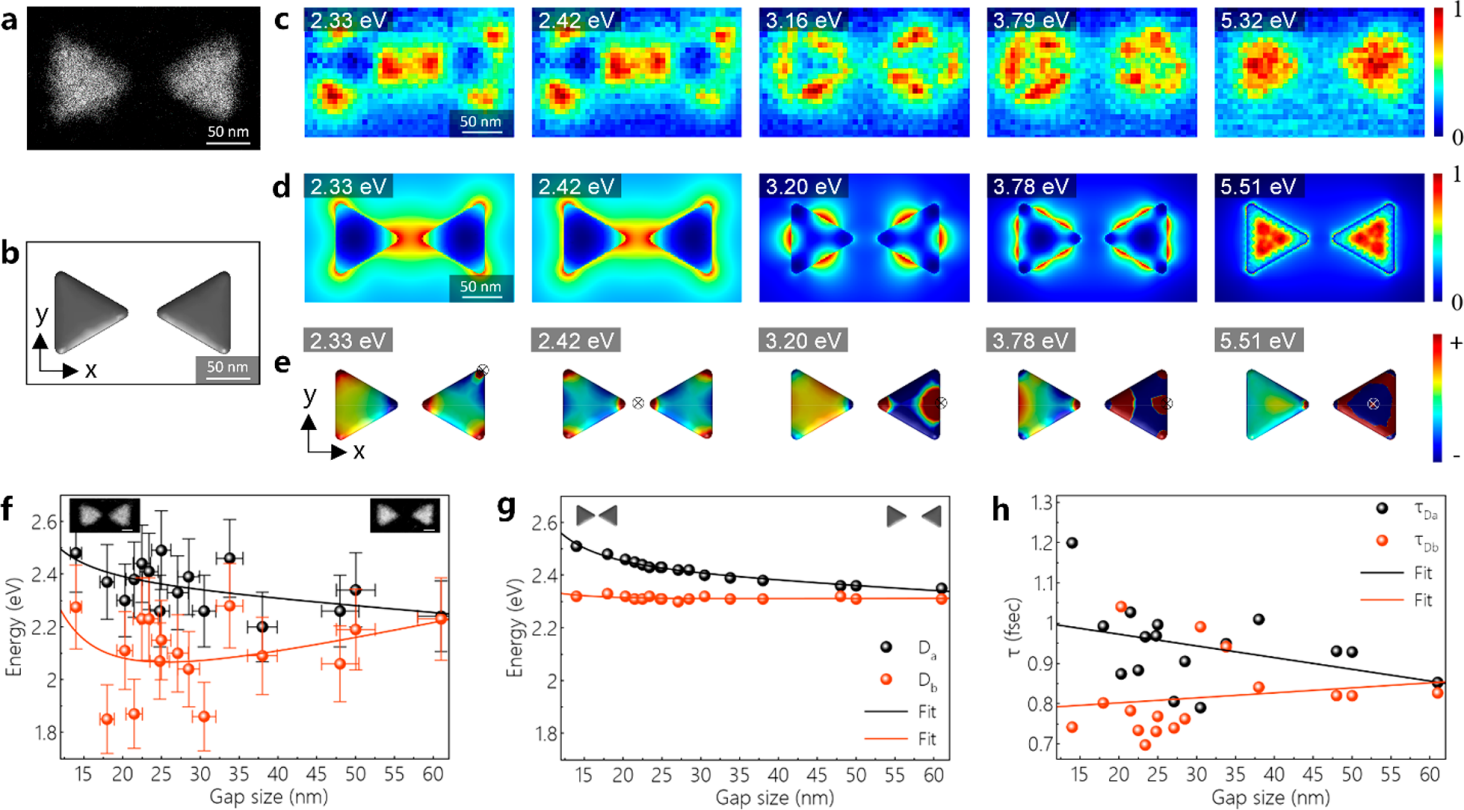
(a, b) HAADF-STEM image of an Al bowtie on graphene and its corresponding model used in BEM simulations. (c, d) Experimental and simulated spatially resolved EEL maps acquired at different energies. (e) Simulated surface-charge distributions of the Al bowtie excited at different energies. The direction of the electron beam is shown by the symbol ⊕. (f, g) Experiment and simulation showing the energies of D_a_ and D_b_ modes depending on the gap size. The scale bars are 50 nm on the HAADF images in panel f. (h) Lifetimes of D_a_ and D_b_ modes depending on the gap size of Al bowties.

We now move on to another structure called bowtie with two Al nanoprisms fabricated on graphene (Figure 5). The HAADF image of an Al bowtie with a gap size of 32 nm and its corresponding model used in BEM simulations are shown in Figure 5a,b. The EEL spectra measured at different positions on the bowtie (Figure S13) show that there are two dipolar LSPR modes excited at energies of 2.42 ± 0.01 and 2.34 ± 0.01 eV (see positions 1 and 4 in Figure S13a). The edge modes excited at 3.16 ± 0.07 and 3.79 ± 0.12 eV are coupled hexapolar and octupolar modes, respectively. Lastly, we observe four breathing LSPR modes at the energies of 4.73 ± 0.09, 5.32 ± 0.18, 6.86 ± 0.04, and 7.60 ± 0.36 eV. With simulated EEL spectra of a bowtie with similar dimensions (Figure S12d), we interpret the experimentally measured LSPRs (Table 1). To verify these LSPRs, we inspect the spatially resolved experimental and simulated EELS maps created at specific energies (Figure S13). The same LSPR modes are further investigated via EFTEM maps for other Al bowties with slightly smaller gap sizes (Figure S14). The EELS and EFTEM maps provide information on the type of LSPRs, but they are not sufficient to distinguish the difference between, for example, antibonding dipole (D_a_) and bonding dipole (D_b_) LSPR modes. The simulated surface-charge distribution in Figure 5e indicates that the D_b_ mode is excited at 2.33 eV, while the LSPR at 2.42 eV corresponds to the D_a_ mode. Figure 5f shows the dependence of the dipolar energy on the gap size. With decreasing gap size, the energy of the D_a_ mode shifts to higher energies as observed in the simulations shown in Figure 5g. Conversely, when shrinking the gap, the D_b_ mode’s energy is reduced, in contrast to the simulated D_b_ mode’s energies remaining nearly constant. We note that the simulations presented in Figure 5 are performed using an effective-medium approach (see the Methods section); therefore, the structures used in the simulations do not involve a graphene membrane and an oxide layer. We assign the difference between experiment and simulation to the variations in the aspect ratio of structures with smaller gaps patterned at high electron doses (see also Figure S2). The lifetime of antibonding (τ_Da_) and bonding (τ_Db_) modes calculated using the Heisenberg uncertainty relation (τ = ℏ/fwhm)^59^ are shown in Figure 5h. The D_a_ modes have a longer lifetime, especially at the bowties with smaller gaps, while the lifetime of D_b_ modes does not exhibit a clear correlation. For bowties with large gaps (>61 nm), both τ_Da_ and τ_Db_ are nearly the same. The simulated surface-charge distribution also ascertains that the higher energy edge and breathing modes at 3.20, 3.78, and 5.51 eV are of bonding-type.

**Figure 6:**
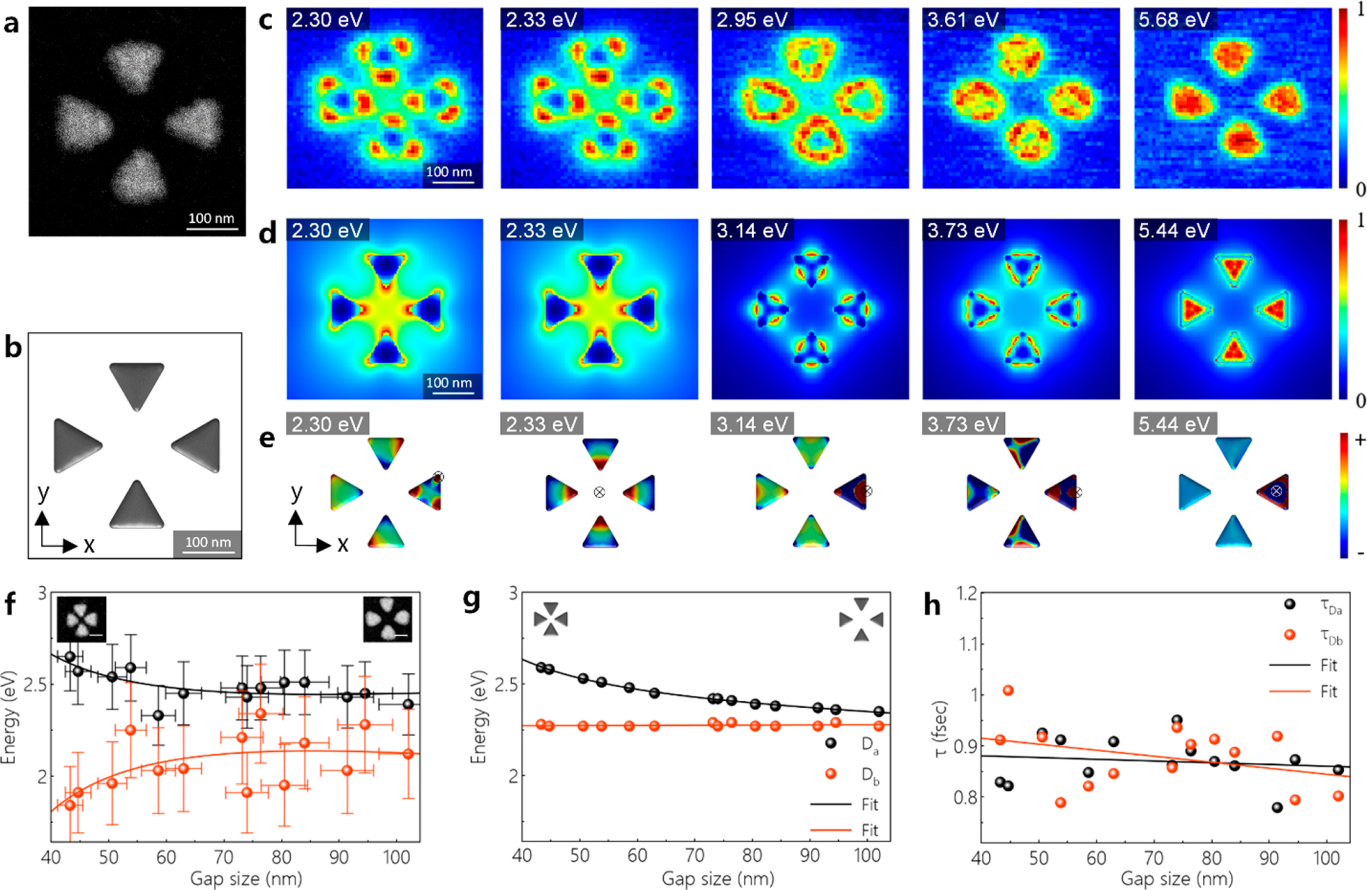
(a, b) HAADF-STEM image of an Al tetramer on graphene and its corresponding model used in BEM simulations. (c, d) Experimental and simulated spatially resolved EEL maps acquired at different energies. (e) Simulated surface-charge distributions of the Al tetramer excited at different energies. The direction of the electron beam is shown by the symbol ⊕. (f, g) Experiment and simulation showing the energies of D_a_ and D_b_ modes depending on the gap size. The scale bars are 50 nm on the HAADF images in panel f. (h) Lifetimes of D_a_ and D_b_ modes depending on the gap size of Al tetramers.

The most complex structure that we have fabricated on graphene is an Al tetramer including four Al nanoprisms (Figure 6a). As this structure has four components, the fabrication of tetramers with smaller gap sizes of <43 nm is demanding. Analogous to the bowties, the tetramers have dipolar, edge, and breathing LSPR modes (see EEL spectra in Figure S15). All of these LSPR modes are confirmed via experimental and simulated EELS maps (see Figure 6c,d) as well as EFTEM mapping (Figure S16). The intensity variations on EFTEM maps are because of the residual nonisochromaticity of the energy filter. The EELS maps acquired on the same sample at 80 kV with a different microscope without a monochromator, involving a faster EELS spectrometer, also support those LSPR modes (Figure S17). The D_b_ and D_a_ modes of the Al tetramer with a gap size of 110 nm are detected at 2.30 and 2.33 eV (Figure 6c–e). Interestingly, after analyzing the simulated surface-charge distributions, we find that the LSPR mode observed at 2.30 eV is a mixed-mode involving both D_b_ and D_a_ modes, whereas the mode at 2.33 eV is only a D_a_ mode. The surface-charge distribution calculated by *x*- and *y*-polarized plane-wave excitation confirms the existence of the D_b_ mode at the energy of 2.30 eV, but it does not show the D_a_ mode, since it cannot be excited by a plane wave (Figure S18). In Figure 6f, the energies of experimental D_a_ modes match well to simulations, but the D_b_ modes are slightly different from the simulated D_b_ modes. The mismatch is likely due to the deviations in the dimensions of nanocavities written at high electron beam doses by electron-beam lithography. In contrast to bowties, there is no clear correlation between the lifetime of dipolar modes and the gap size. For both nanocavities, the quality factor *Q* (*Q* = *E*/Δ*E*, where *E* is the resonance energy of the dipolar mode, and Δ*E* is the line width^60^) of D_a_ modes (in the range of ∼3–4.5 eV) is higher compared with the D_b_ modes’ *Q*, which is in the range of ∼2–3.5 eV (Figure S19). While *Q* of the D_a_ mode is enhanced when decreasing the gap size of both nanocavities, *Q* of the D_b_ mode is almost independent of gap size. Since the emitter–cavity interaction is enhanced with high *Q*, the bowties with smaller gap sizes show a great promise for the realization of strong coupling, while tetramers are more favorable for a charge transfer due to their wide range of D_a_ modes that can be excited within the hotspot and at the poles of nanoprisms. Compared to simulated EEL spectra of the monolayer graphene, showing a clear low-energy plasma excitation (π plasmon) at 5.26 eV, the π plasmon peak at the experimental EEL spectra is much broader, and it shows a shoulder at ∼4.5 eV (Figure S20a,b). This is an indication of an amorphization of graphene probably due to electron beam damage.^61^

### Intermediate Coupling in the Nanocavity–NC System

The Pt NCs (>4.6 nm) show a plasmon absorption in the UV spectrum.^62,63^ Since the LSPR energies of metal nanoparticles blue-shift with decreasing particle size,^64,65^ the LSPRs of smaller Pt NCs (<4.6 nm) are expected to be in the UV range. Contrary to this argument, Bornacelli et al. demonstrate that 1.6 nm Pt NCs do not exhibit a plasmon absorption in the UV range, but they rather form uncorrelated electron–hole pairs leading to a superlinear PL enhancement.^62^ Another study performed by Wieghold et al. indicates that the LSPRs of 1 nm Pt NCs can be activated by placing them on a Au plasmonic support resonating at 2.29–2.38 eV.^51^ Briefly, the existence of LSPRs and excitonic states of atomically small Pt NCs is not clear and not yet well-understood. Similar to Wieghold et al.,^51^ we do not observe a clear dipole excitation in the individual Pt NCs with an average size of 1.33 nm (Figure S21c,d). Unlike in the literature, the dipolar energies of 1.33 nm Pt NCs are measured to be in the visible spectrum. The LSPRs of plasmonic nanoparticles and the exciton energies of semiconductor quantum dots are red-shifted with increasing particle size.^65,66^ Similarly, we observe a red-shift in the LSPRs of the larger Pt NCs. Not only the particle size but also the dielectric medium leads to a shift in LSPRs.^67,68^ We thus assume that the dipolar energies of Pt NCs are red-shifted toward the visible spectrum because of the formation of an amorphous PtO_*x*_ surrounding the metallic Pt (see the EDS spectrum of a Pt NC in Figure S7e). Moreover, the formation of an amorphous carbon layer covering the Pt NCs during the EELS line scan also induces a red-shift in the LSPRs of Pt NCs (Figures S22 and S23).

**Figure 7:**
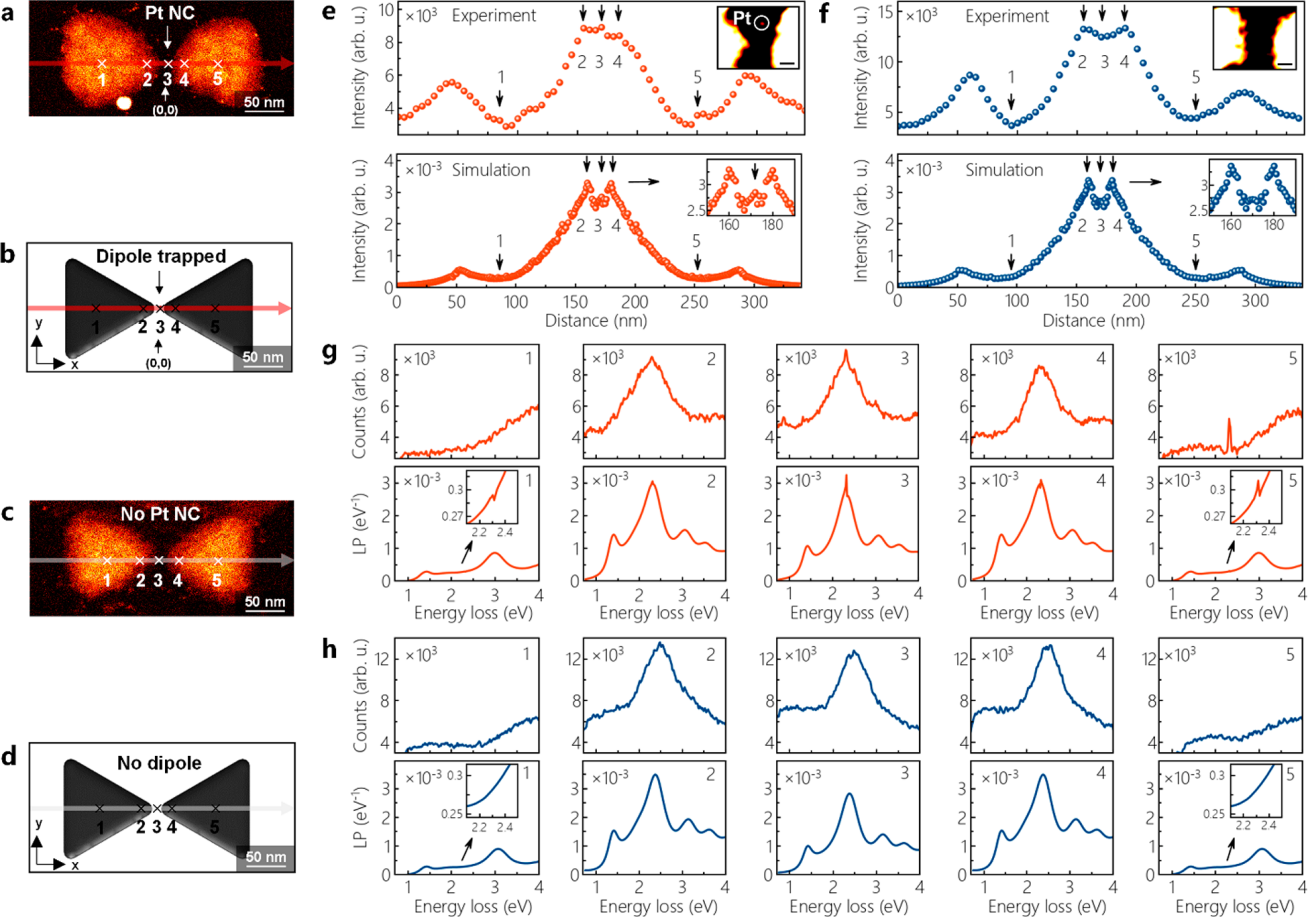
(a, b) HAADF-STEM image of an Al nanocavity with a Pt NC (∼1.18 nm) trapped within the plasmonic hotspot and its corresponding model used in BEM simulations. The Pt NC and dipole are placed to the (*x*, *y*) coordinates of (2 nm, 3.1 nm) in panels a and b. The center of the nanocavity is at the coordinate of (0, 0). (c, d) HAADF-STEM image of an Al nanocavity without a Pt NC and its corresponding model used in BEM simulations. (e, f) Experimental and simulated intensities of a dipolar mode excited along the red (for Al bowtie with a Pt NC) and blue (for Al bowtie without a Pt NC) transparent lines in the panels a–d. The intensity is averaged over an area of 0.1 eV on dipolar modes in the experimental and simulated data. The insets show the close-up HAADF images of the hotspots in the nanocavities shown in panels a and c. The images are Gaussian blurred (radius 1 px) and in false color. The scale bars are 8 nm. (g, h) Experimental and simulated EEL spectra obtained at the positions marked on panels a–d, respectively. The background in experimental EELS data is subtracted after a power-law fitting.

A Pt NC trapped within the plasmonic hotspot of an Al bowtie with a ∼14 nm gap and its corresponding model are shown in Figure 7a. Here, the Pt NC pointed at by a white arrow is closer to the Al nanoprism on the right side. Figure 7c,d shows another Al bowtie with a ∼14 nm gap but without a Pt NC trapped within the hotspot and its corresponding model used in BEM simulations. The averaged intensities of dipolar modes shown in Figure 7e,f are measured from an energy window of 0.1 eV on the EEL spectra acquired along the red and blue arrows on the HAADF images and the models (Figure 7a–d). Here, the intensity of dipolar modes is found to be higher at the edges of nanoprisms due to the D_b_ mode excited at the distances of 50 and 290 nm as well as at the poles (155 and 185 nm) and hotspot (170 nm) due to the excitation of the D_a_ mode. When the electron beam is in close proximity to the Pt NC, the intensity of the D_a_ mode reaches the maximum as observed in the simulations (see position 3 in Figure 7a–f). In the case that a Pt NC is absent within a hotspot, we do not observe a strong enhancement at the dipolar mode (see Figure 7c,d,f and Figure S24). As reported earlier, the dipole LSPR of the Pt NCs with a diameter of 1 nm can be activated by placing them nearby a plasmonic support.^51^ The sharp peak that appears on the D_a_ mode is thus attributed to the energy transfer from the D_a_ mode of the bowtie to the Pt NC. Additionally, a weak dipole intensity is rising when the electron beam is located at the center of an Al nanoprism (see positions 1 and 5 in Figure 7a–f). To figure out the mechanism behind the enhancement of the dipolar intensity, we compare the experimental and simulated EEL spectra shown in Figure 7g. Since a sharp peak appears directly on the D_a_ mode of the bowtie (see position 3 in Figure 7g), we set the energies of both the D_a_ mode in the bowtie and dipole mode in the Pt NC trapped within the hotspot to 2.32 eV in the BEM simulations. Notably, the EEL spectra acquired at position 3 and loss probabilities (LPs) calculated at the same position show a strong increase in the intensity (Figure 7g). Here, we find that the strong enhancement stems from the coupling between the D_a_ mode of the bowtie and the dipole mode of the Pt NC. When there is no Pt NC within the hotspot, the intensity of the D_a_ mode at position 3 is lower compared with the intensities at positions 2 and 4 (Figure 7c,d,h). BEM simulations show that the position of the Pt NC within the hotspot is critical for the realization of dipole–dipole coupling (Figures S25 and S26). The intensity is reduced when the Pt NC is placed far from the center of the hotspot along the *y*-axis while the *x*-position is stationary (Figure S25). If the Pt NC is displaced along the *x*-axis (its position on the *y*-axis is stationary), the intensity reaches the maximum at positions where the Pt NC is closer to one of the Al nanoprisms in the bowtie (Figure S26). To reveal the existence of different dipole–dipole coupling regimes, we fitted the experimental EEL spectra to a coupled oscillator model (Figure S27). In the coupled oscillator model, the resonance energies of the uncoupled D_a_ mode in the bowtie (ω_cp_) and the uncoupled dipole mode in the Pt NC (ω_pp_) are the same (ω_cp_ = ω_pp_ = 2.32 eV). The coupling strength *g* is found to be 10 meV from the fitting for this hybrid system (Figure S27). The criterion for the strong coupling is given by 2*g* > (γ_cp_ – γ_pp_)/2 or 2*g* > (γ_cp_ + γ_pp_)/2,^17,69,70^ where γ_cp_ (429 meV) and γ_pp_ (5.8 meV) are the line widths of the D_a_ mode and the dipole in the Pt NC (see the Methods section for details). The criterion of 2*g* > (γ_cp_ + γ_pp_)/2 is more strict and requires a higher *g* to fulfill. As this system does not fulfill these criteria, the dipole–dipole coupling is not in the strong coupling regime. The coupled-oscillator model simulations of EEL spectra suggest that the strong coupling might be visible for *g* ≥ 110 meV (Figure S28). In addition to the strong enhancement at 2.32 eV, we detect a sharp peak at 2.31 eV when the e-beam is placed at the center of a nanoprism in both the experimental structure and the model (see position 5 in Figure 7a,b,g). As shown in the EELS maps of bowties in Figure 5c–e, a bonding breathing mode is excited at this position. The existence of radial breathing modes, which can couple to the light, in both Al nanodisks and nanoprisms has been reported earlier.^71−74^ The peak at 2.31 eV originates from the coupling of the dipolar mode of the Pt NC to the bonding breathing mode of the Al nanoprism. This peak is not visible at position 1 (center of the other nanoprism on the left side), since the Pt NC is closer to the nanoprism located on the right side (Figure 7a,b,g). The steep peak at 2.31 eV is observed only when a bonding-type breathing mode of the bowtie couples a dipole LSPR of the Pt NC within the nanocavity (Figure 7g,h). We thus rule out the existence of both excitons and uncorrelated electron–hole pairs in the Pt NCs with an average size of ∼1.33 nm. Unlike individual Pt NCs that do not present any clear plasmon absorption, the Pt NCs confined within the plasmonic hotspots indicate an enhanced dipole absorption (see position 3 in Figure 7g), while there are Fano dips appearing at positions 1 and 5 (Figure 7g). This hybrid system is thus in the intermediate coupling regime enabling the energy transfer from the dipole LSPR of the Al bowtie to the Pt NC.^75^

As the Pt NCs with varying sizes are randomly distributed around the Al plasmonics, we cannot control their positions within the hotspots. For this reason, a detuning cannot be obtained experimentally. Hence, we inspect the coupled-oscillator-model simulations of EEL spectra for a possible detuning obtained by varying the resonance energy of the dipole (Figure S29). Here, ω_pp_ is varied from 1.6 to 3.04 eV, while ω_cp_ is 2.32 eV. Similar to the system in Figure 7a, the criterion for the strong coupling is not fulfilled for *g* = 10 meV. Instead, we observe a clear peak splitting for *g* = 160 meV since 2*g* > (γ_cp_ + γ_pp_)/2. Here, a Rabi splitting given by  is ∼240 meV. The minimum Ω of ∼240 meV is observed at a detuning of 0 meV. Since the system fulfills the two criteria 2*g* > (γ_cp_ + γ_pp_)/2 and Ω > (γ_cp_ + γ_pp_)/2, the anticrossing behavior, which is the signature of strong coupling, is explicitly visible in the counterplot of the EEL spectra (Figure S29c).

Besides changing the resonance energy of the dipole trapped within the nanocavity, we change the bowtie gap size (from 6 to 64 nm) to obtain the detuning (Figure S30). Here, ω_cp_ is reduced from 2.364 to 2.175 eV by increasing the gap size of the bowtie while ω_pp_ = 2.253 eV. In this system, both 2*g* and Ω are higher than (γ_cp_ + γ_pp_)/2 for *g* = 160 meV. Hence, the Rabi splitting and the anticrossing are apparent in this strongly coupled system (Figure S30a,b).

Lastly, we try to obtain a detuning by changing the location of the dipole within the hotspot of the bowtie (Figure S31a). Here, ω_cp_ varies from 2.336 to 2.261 eV when the bowtie is excited with an electron beam located at different coordinates ranging from (*x* = 3 nm, *y* = 0 nm) to (*x* = 3 nm, *y* = 80 nm). The dipole with a constant resonance energy (ω_pp_ = 2.32 eV) is located at the same coordinates to reveal the coupling behavior of the hybrid system. For *g* = 10 meV, the system is in the weak coupling regime (Figure S31b). With an increase of *g* to 160 meV, 2*g* and Ω become higher than (γ_cp_ + γ_pp_)/2. In this way, a Rabi splitting emerges, and the system passes to the strong coupling regime (Figure S31c). Since the uncoupled resonance energy of the D_a_ mode does not vary strongly, the anticrossing behavior is barely visible for this system (Figure S31d).

The strong enhancement of the dipolar intensity is further shown at another experimental structure (Figure S32). Here, the bowtie has a gap size of 68 nm, and the Pt NC is closer to the Al nanoprism located on the left side of the bowtie. Similar to the previous system shown in Figure 7, the maximum intensity is observed at the position where the Pt NC exists (see position 3 in Figure S32a,c,f,g). In the BEM simulations, the resonance energies of the D_a_ mode excited at the center of the hotspot and the dipole are set to 2.26 and 2.252 eV, respectively. As shown in Figure S31, the energy of the D_a_ mode excited at the center of the hotspot is higher compared to the energy of the D_a_ mode excited at a position far from the center. Thus, the resonance energy of the D_a_ mode is 2.252 eV at position 3, where the Pt NC is located. Since the Pt NC is closer to the Al nanoprism located on the left side, a small sharp peak appears when the breathing mode of this nanoprism is excited (see position 1 in Figure S27a,c,f,g). To elucidate the coupling regime, we fit the experimental EEL spectra taken from positions 3 to the coupled oscillator model with the parameters of ω_cp_ = ω_pp_ = 2.252 eV (Figure S33). As the criterion of 2*g* > (γ_cp_ – γ_pp_)/2 is not fulfilled for *g* = 55 meV obtained from the fitting, this system is also not in the strong coupling regime. Using a similar model system, we perform coupled-oscillator-model simulations of EEL spectra to assess its coupling regime (Figure S34). The EEL spectra from a zero-detuned coupled system do not present a clear anticrossing behavior due to the small energy variations in the D_a_ mode excited at different positions within the nanocavity.

Similar to the previous hybrid system shown in Figure 7a, the intensity of the Pt NC is enhanced by the D_a_ mode of the bowtie (Figure S32). We hereby show that these hybrid systems are in the intermediate coupling regime as both of them enable enhanced absorption via energy transfer from the D_a_ LSPR of the bowtie to the Pt NC. As noted earlier, the individual Pt NCs far from the nanocavities do not support the strong plasmon absorption. However, when they are trapped within the hotspots of patterned Al nanostructures, we observe a strong enhancement in their dipolar LSPR mode, which couples to the D_a_ and breathing modes of the Al plasmonics. Thus, the patterned Al nanostructures on graphene enabling the activation of LSPRs in the Pt NCs can be used to improve the catalytic activity of Pt NCs via the formation of plasmon-mediated LSPRs and hot electrons.

## Conclusion

We report an approach enabling the fabrication of low-cost plasmonic nanocavity arrays at a large area on CVD-grown monolayer graphene. BEM simulations of EEL spectra show that monolayer graphene is a good substrate for Al plasmonics. The Al bowtie and tetramer nanocavities support LSPRs at the visible and UV region of the electromagnetic spectrum. Both structures display antibonding and bonding dipole modes, which are excited within the hotspot of the nanocavity and poles of Al nanoprisms. Interestingly, the tetramer allows the excitation of both antibonding and bonding dipole modes simultaneously at the poles of Al nanoprisms. Controlling the gap size of both bowties and tetramers enables obtaining tunable dipolar modes in a wide energy range. Consequently, the bowties with a high *Q* show great promise for weak, intermediate, and strong coupling, while the tetramers with a lower *Q* have a high potential to be used for hot carrier generation due to their wide-range antibonding dipole modes that can be excited at the poles of nanoprisms and within the hotspot. In addition to the fabrication and characterization of Al plasmonics on graphene, we report on how to trap Pt NCs within the plasmonic hotspots of nanocavities on atomically clean graphene by thermal annealing at 300 °C for 15 min. The hybrid nanocavity–NC systems also exhibit an intermediate coupling enabling enhanced dipole absorption in Pt NCs due to the energy transfer from the antibonding dipole of the nanocavities to the Pt NCs. Notably, we further reveal the coupling between the dipole mode of a Pt NC and the bonding breathing mode of an Al bowtie. In light of these findings, our approach provides a route for the fabrication of plasmonics and electronic devices on ultrathin and flexible substrates. We propose that Al bowties, showing great promise for weak and strong coupling, and Al tetramers, having high potential for hot carrier generation and light absorption, can find applications in quantum technologies (e.g., quantum information processing), plasmon-mediated catalysis, and surface-enhanced fluorescence.

## Methods

### Sample Preparation

Plasmonic nanocavities were fabricated on monolayer graphene grown on a Cu foil (Graphenea Inc.) by electron-beam lithography (see Figure S1). The CVD graphene on Cu foil (∼4 mm × 4 mm) was first cleaned with acetone and then rinsed in isopropanol. The graphene/Cu stacks were then placed on a flat Si chip and coated with an 80–90 nm thick PMMA (2% PMMA 950k in anisole) by a spinner running at 6000 rpm (acceleration rate of 2000 rpm/s) for 35 s. Here, the Si chip plays a critical role in keeping the Cu foil flat during the lithography process. Following the spin-coating of the resist, the samples placed on a hot plate were heated at 160 °C for 4 min. The patterning of the resist was carried out by a Raith eLine with a 7.5 μm objective aperture and an acceleration voltage of 15 kV. The working distance, the measured beam current, and the areal dose were 9 mm, 19.6 pA, and 1200 μC/cm^2^, respectively. The exposed resist was then developed in methyl isobutyl ketone (MIBK)/isopropyl alcohol (IPA) solution (3:1) at 0 °C for 30 s. The samples with an exposed resist were cooled by a custom design cooler enabling chilling in a nitrogen environment. The samples developed were dried by a nitrogen spray gun. After the development of samples, 30 nm thick aluminum (99.99% purity) was deposited on them by a thermal evaporator (Univex 1) with a base pressure of ∼1.6 × 10^–6^ mbar. The deposition rate of Al was kept at ∼2 Å/s (on a quartz crystal microbalance). For the metal lift-off, the sample was immersed in *N*-methyl-2-pyrrolidon (NMP), heated at 60 °C for ∼30 min, rinsed with acetone and isopropanol, respectively, and finally dried by the nitrogen spray gun. Before the transfer of Al nanocavities fabricated on a graphene/Cu stack, a 10 nm thick Pt film was deposited on a Au TEM grid with a carbon film with holes (Quantifoil) by a sputter coater (Leica). The position of the TEM grid on the sample was aligned using a home-designed micromanipulator. To create adhesion between the Quantifoil and graphene, one drop of isopropanol was applied. The sample was then placed on a 10% ammonium persulfate (APS) solution for ∼3 h to etch the Cu foil away. Subsequently, the TEM grid carrying the nanocavity/graphene stack was rinsed with isopropanol. SEM images of the samples after fabrication and transfer processes were recorded using a Zeiss Gemini DSM 982 instrument with a cold field-emission gun and an in-lens detector at an acceleration voltage of 5 kV. The working distance was set to 14 mm in the measurements.

### Atomic Resolution HRTEM and STEM Measurements

HRTEM measurements were performed by a JEOL ARM200F TEM instrument with a cold field-emission gun and a postspecimen spherical aberration corrector (*C*_s_) at under-focus conditions. All HRTEM images were acquired at the acceleration voltage of 80 kV. For the atomic resolution HAADF imaging, we used a JEOL ARM200F FEG-STEM/TEM instrument equipped with a cold field-emission gun, a CEOS *C*_s_-corrector (CEOS GmbH), and a Gatan GIF Quantum ERS electron energy-loss spectrometer. Atomic resolution images of Pt NCs were recorded at an accelerating voltage of 200 kV when the camera length and collection semiangle range were 6 cm and 76–250 mrad, providing high-angle annular dark-field (HAADF) imaging conditions, respectively.

### EELS, EFTEM, and EDS measurements

The sub-electron-volt–sub-angstrom microscope (Zeiss SESAM) equipped with a Schottky field-emission gun, an electrostatic OMEGA-type monochromator, and a high-dispersion and high-transmissivity MANDOLINE filter was used for low-loss EELS and EFTEM measurements. All of the measurements were done at an acceleration voltage of 200 kV. The energy resolution, energy dispersion, and EELS collection semiangle were 0.17 eV, 0.015 eV/px, and 0.7 mrad, respectively. The pixel dwell time was set to 3 s in the line scans, while it was set to 0.5 s in the EELS maps. The EELS maps were extracted by summing over an energy window of 0.2 eV. The EELS maps were processed with a multivariate weighted principal component analysis (PCA) routine.^76^ A Gaussian fitting was applied to EEL spectra in order to define the peak position of LSPRs whereas a Lorentzian fitting was used to extract the full width at half-maximum (fwhm) for calculating plasmon lifetimes. In the EFTEM maps of nanocavities acquired in the energy range of 0–16 eV and at the energy step size of 0.1 eV, we used the smallest energy slit of 0.23 eV. The integration time was 6 s during EFTEM mapping. The JEOL ARM200F TEM instrument with a postspecimen spherical aberration corrector (*C*_s_), a Gatan GIF Quantum ERS electron energy-loss spectrometer, and an EDS spectrometer was also used to perform EFTEM thickness and EELS measurements at 80 kV. Here, the energy resolution, dispersion, and pixel time were set to 0.5 eV, 0.01 eV/px, and 0.01 s, respectively. The EFTEM thickness measurements were done using an energy-slit width of 10 eV and an exposure time of 2.44 s. The EDS measurements were performed under the same conditions at 80 kV.

### Electromagnetic Simulations

Boundary element method simulations of EEL spectra were performed via a Matlab toolbox (MNPBEM).^77^ The dielectric functions of Al, monolayer graphene, and monolayer h-BN were taken from McPeak et al., Nelson et al., and Beiranvand et al.^78−80^ The dielectric constant of 4.25 was used for SiN in the simulations.^25^ The dimensions of the structures were set to the dimensions observed in the experimental data. The bowtie structures in the simulations were constructed using two equal nanoprisms with an edge length of 80 or 120 nm and a height of 20 nm. For simplicity of complex simulations (e.g., bowtie on the membrane and tetramer on the membrane), an effective-medium approach was employed. Thus, the existence of a membrane and an oxide layer covering the Al nanoprism was disregarded for bowtie and tetramer structures. An electron-beam excitation with beam energies of 80 and 200 keV was used in the simulations. BEM simulations of EEL spectra were performed in the energy range of 0.73–8 eV with a 300 mesh. The EEL simulations of a bowtie coupled to a Pt NC were carried out by placing a sphere with a radius of 2.51 nm within the hotspot of bowties. The resonance energy of Pt NCs was varied by changing the dielectric constant of a 0.5 nm thick cover layer around the Pt NC. The complex dielectric function of the Pt NC was approximated by a Lorentz model with the following parameters: high-frequency dielectric constant ϵ_∞_ = 1, oscillator strength *f* = 0.8, ω_0_ = 2.356 eV, and γ_0_ = 0.08.^17^

### HRTEM and STEM Image Simulations

HRTEM and HAADF image simulations were carried out using QSTEM software with parameters corresponding to the experiments.^81^ The parameters used for HRTEM simulations were as follows: a spherical aberration coefficient of 1 μm, accelerating voltage of 80 kV, and a defocus of −2.5 nm. For HAADF-STEM simulations, the following parameters were used: chromatic aberration coefficient of 1 mm, spherical aberration coefficient of 1 μm, accelerating voltage of 200 kV, and convergence angle of 20.4 mrad. The HAADF detector angle range was set to the experimental range of 67–250 mrad.

### Coupled Oscillator Model

The uncoupled oscillators assumed in the coupled oscillator model are the antibonding dipole LSPR of the bowtie and the dipole LSPR of the Pt NC. Here, both dipole LSPRs of the bowtie and the Pt NC are described as damped harmonic oscillators, which represent the polarization of these structures.^82^ Both of these structures are coupled via the electric near-field with a coupling strength (*g*). By solving the equations of motion for these two oscillators, the extinction of two coupled oscillators at the frequency of ω is obtained.^17^ The experimental EEL spectra were fitted to this model in order to estimate the coupling strength.
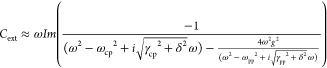
1Here, ω_cp_ and ω_pp_ are the resonance frequencies of the D_a_ mode of the bowtie and the dipole mode of the Pt NC, while γ_cp_ and γ_pp_ are the line widths of these uncoupled oscillators (the D_a_ mode and the dipole mode of the Pt NC), respectively. In the coupled oscillator model, we used the line widths obtained from the simulated EEL spectra of the uncoupled bowtie and the Pt NC and added the instrumental broadening in the fitting. Here, the instrumental broadening δ is 80 meV, while γ_cp_ = 429 meV and γ_pp_ = 5.8 meV for all of the simulations.

## Supporting Information Available

Schematic of the sample fabrication; additional SEM, STEM, TEM, EDS, EELS, and EFTEM data; additional electromagnetic simulations; and coupled-oscillator-model simulations of EEL spectra (PDF)
