# Supplementary material for: Hybrid Graphene-Supported Aluminum Plasmonics
Source: ACS Nano. 2022 Jul 29;16(8):11931–43. doi: 10.1021/acsnano.2c01730 (PMC9413403; doi:10.1021/acsnano.2c01730)
Supplement: Supplementary file 1 — nn2c01730_si_001.pdf [file nn2c01730_si_001.pdf]

Supporting Information:

Hybrid graphene-supported aluminum plasmonics

Kenan Elibol* and Peter A. van Aken

Stuttgart Center for Electron Microscopy, Max Planck Institute for Solid State Research Heisenbergstr. 1, 70569 Stuttgart, Germany.

Corresponding Author
*E-mail: k.elibol@fkf.mpg.de

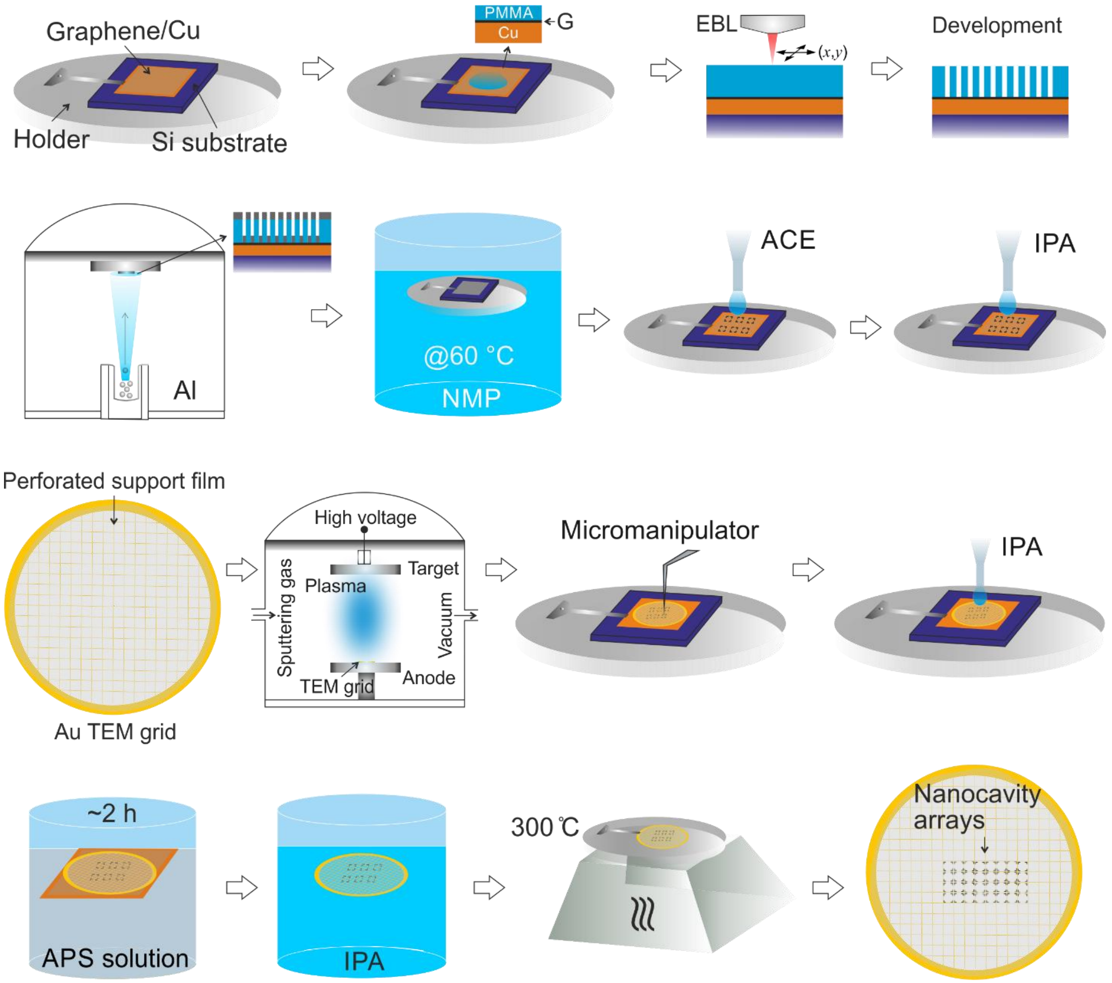

Supplementary Figure S1. Schematic of the sample fabrication. (a) Graphene grown on a Cu foil is placed on a flat Si substrate and fixed on a sample holder using a copper clamp. (b) Spin coating of 80-90 nm-thick positive tone PMMA and subsequent heating to 160℃ holding the temperature for 4 min. (c) Patterning of PMMA by a 15 kV electron beam. (d) Development in MIBK/IPA (3:1) at 0℃ for 30 s. (e) Thermal evaporation of Al onto the patterned surface. (f) Metal lift-off in NMP heated at 60℃ for ~30 min. (g,h) Rinsing the structures in acetone and isopropanol, respectively. (i) Au TEM grid covered with a perforated support film (quantifoil). (j) Sputter coating of a 10 nm-thick (on quartz microbalance) Pt layer on the support membrane covered on the TEM grid. (k) Positioning of TEM grid on the nanocavity arrays fabricated on the graphene surface. (l) Putting a drop of isopropanol to create adhesion between the quatrefoil and graphene. (m) Removing of Cu by etching in a 10 % APS solution for ~2 hours. (m) Rinsing the sample in isopropanol after etching the Cu foil. (e) Thermal heating of the TEM grid at 300℃ in air for 15 min. (p) Schematic of the TEM grid with nanocavity arrays fabricated on a ML graphene membrane.

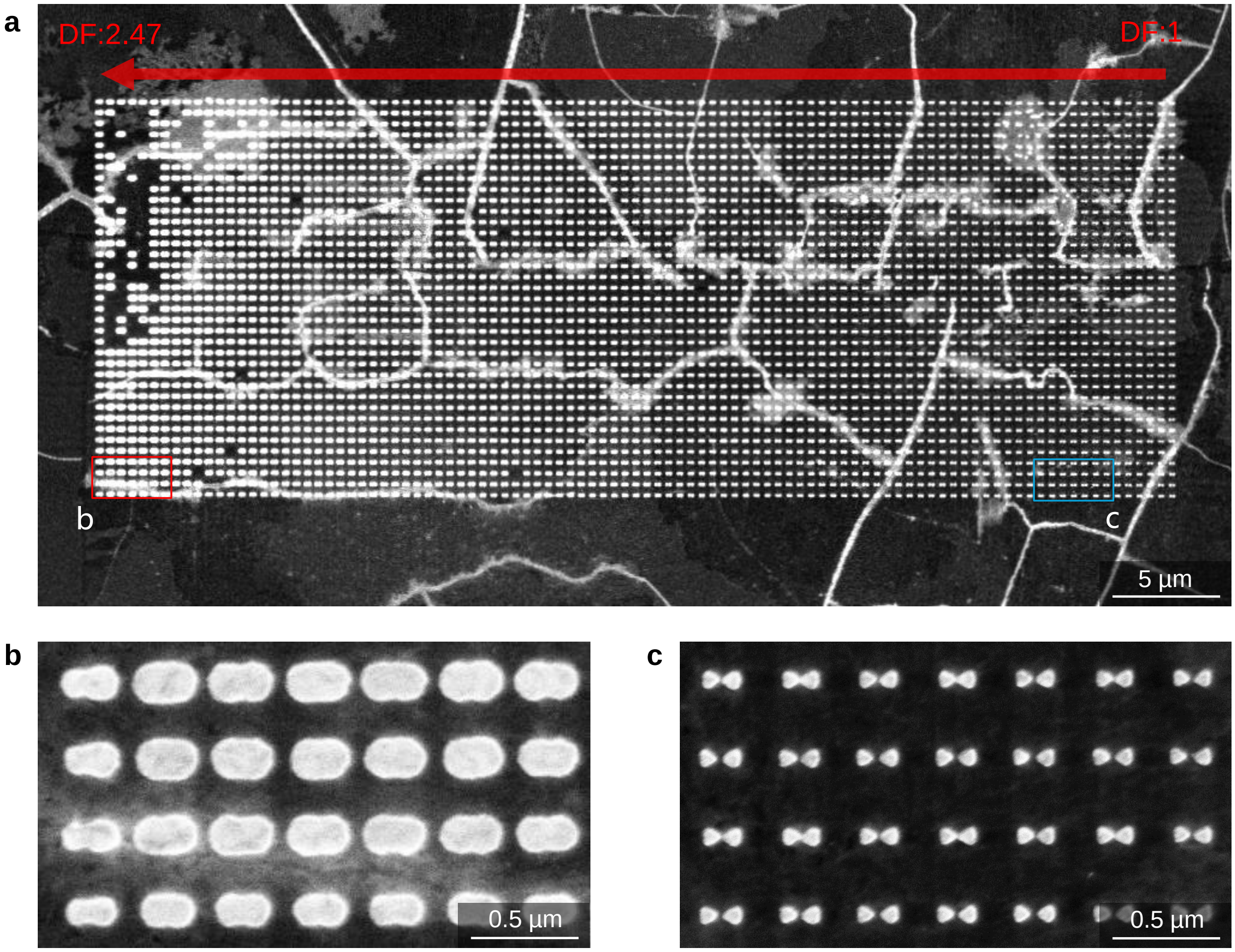

Supplementary Figure S2. SEM image of an Al bowtie array fabricated on CVD-grown graphene on a Cu foil. The base dose used during patterning is 1300 µC/cm^2. The dose factor is enhanced from 1 to 2.47 to control the gap size of nanocavities. Enlargements of areas marked with (b) red and (c) blue rectangles on panel (a).

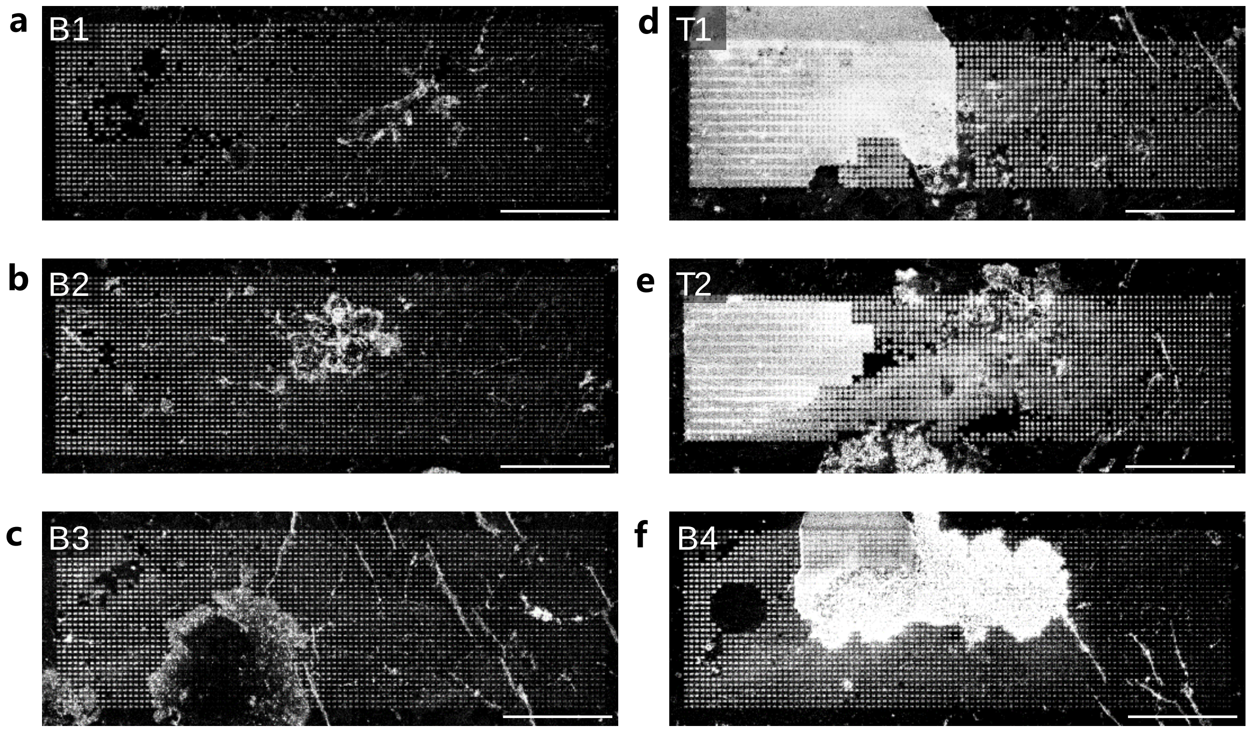

Supplementary Figure S3. (a-e) SEM images of the bowtie (B1, B2, B3, B4) and tetramer (T1, T2) nanocavity arrays on graphene/Cu. The scale bars are 10 μm.

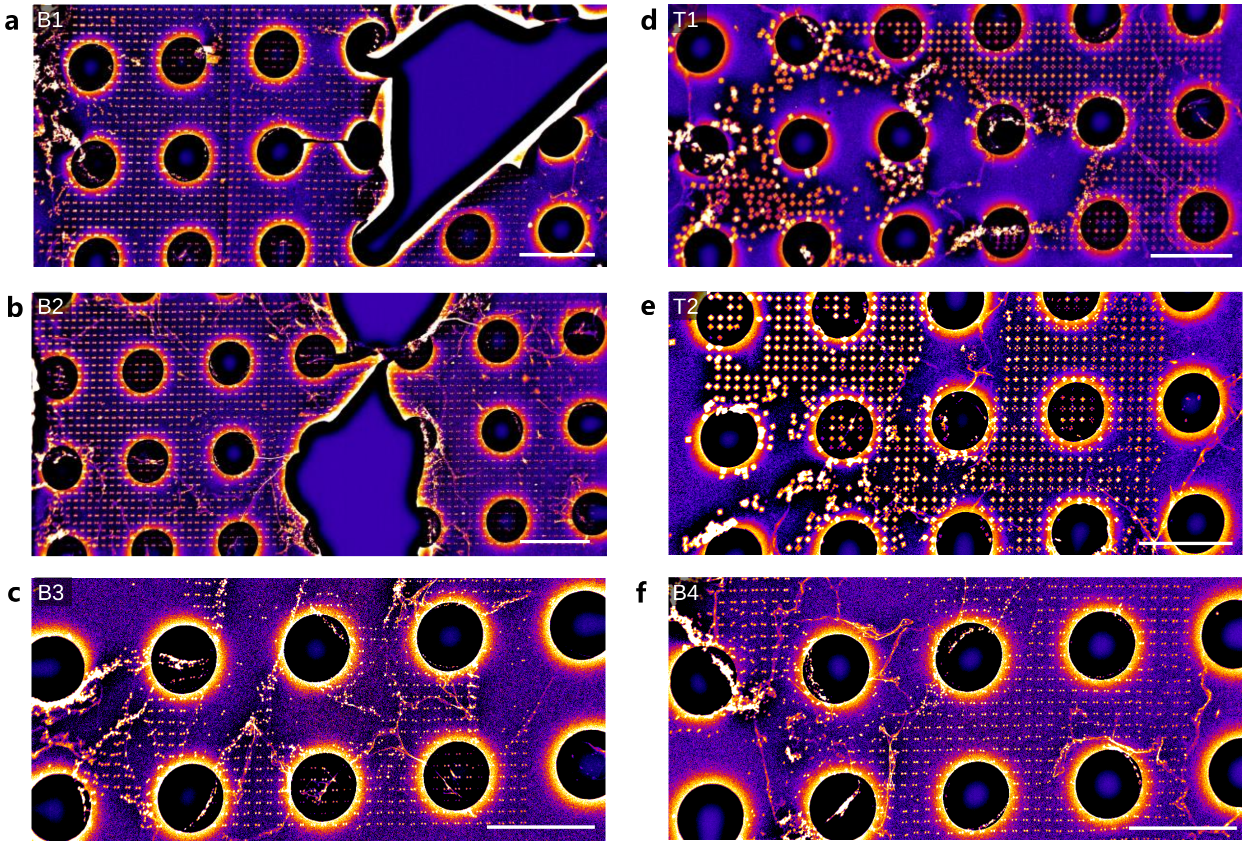

Supplementary Figure S4. (a-e) HAADF-STEM images of the bowtie (B1, B2, B3, B4) and tetramer (T1, T2) nanocavity arrays transferred on a Au TEM grid with quantifoil. The scale bars are 5 µm. The images are background subtracted and false colored.

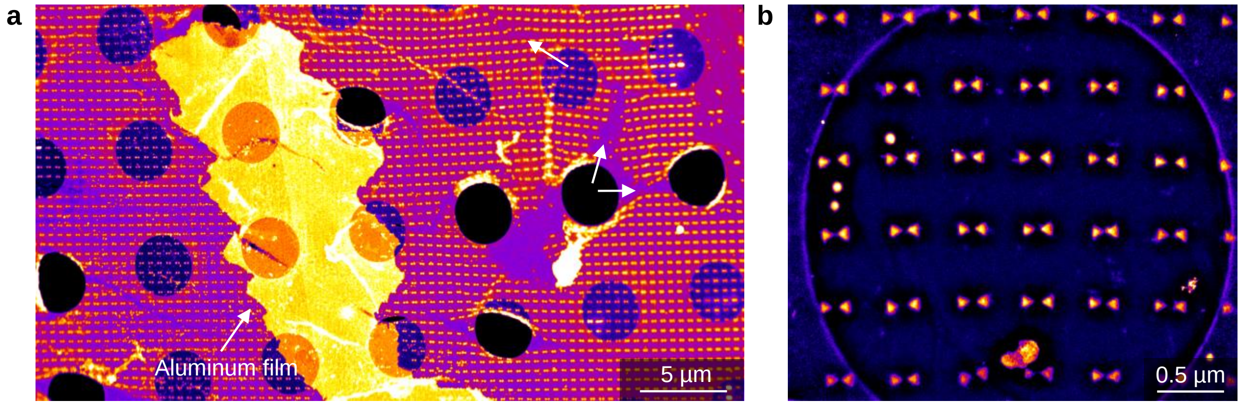

Supplementary Figure S5. (a) HAADF-STEM image of an Al bowtie nanocavity array transferred on a TEM grid. (b) Close up HAADF-STEM image of one of the membranes including Al nanocavities. The image is background subtracted and false colored.

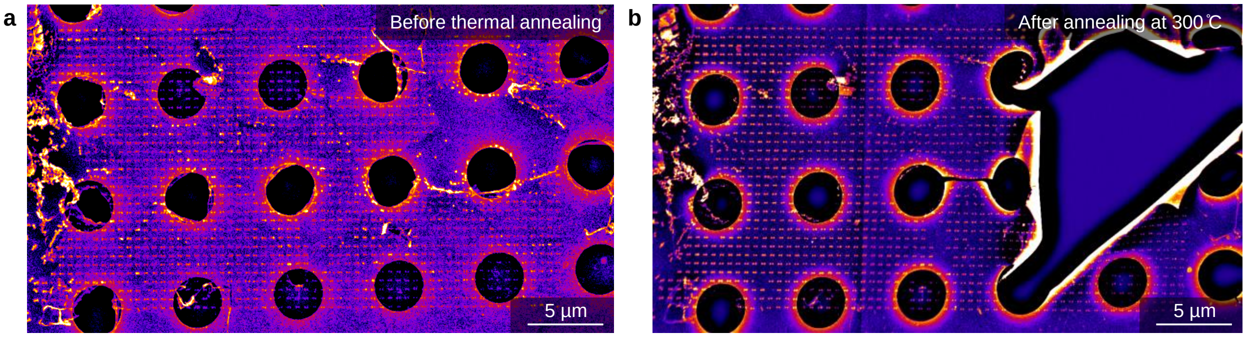

Supplementary Figure S6. (a) SEM and (b) HAADF-STEM images of the Al bowtie array (see B1 in Figure 1b and c in the main text) before and after thermal annealing. The images are background subtracted and false colored.

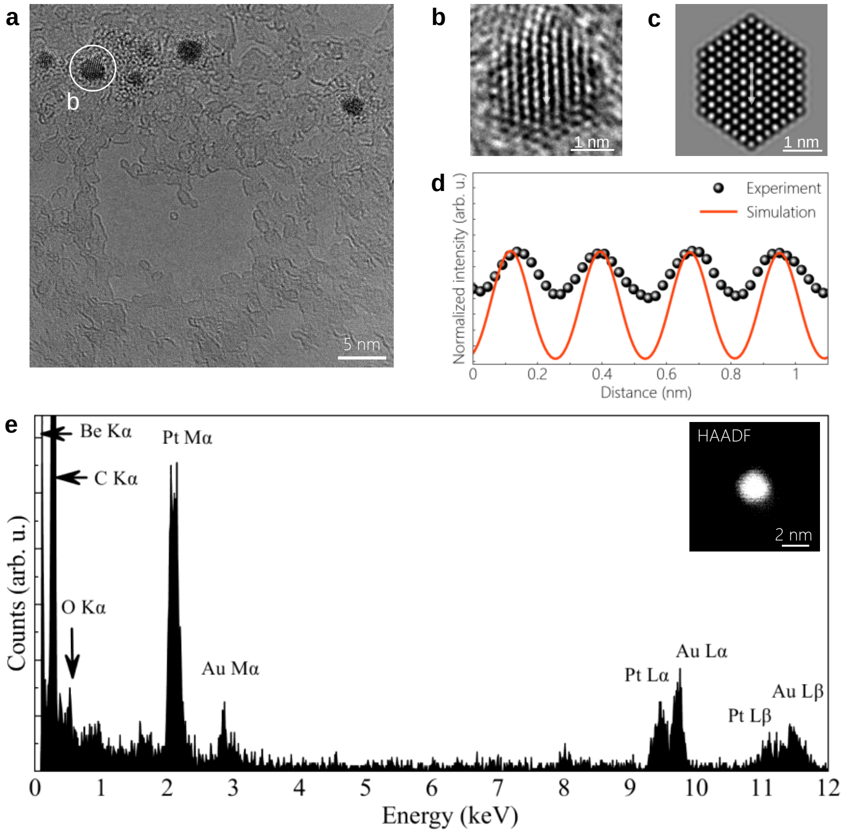

Supplementary Figure S7. (a) HRTEM image of Pt NCs formed on graphene after thermal heating at 300℃ in air for 15 min. (b,c) Close up image of the Pt (110) NC marked on panel a and its corresponding simulated HRTEM image. (d) Line profiles taken along the white arrows on the experimental and simulated HRTEM images of the Pt (110) NC. (e) EDS spectrum recorded on a Pt NC. The inset shows the HAADF-STEM image of the analysed Pt NC. The peaks corresponding the Be and Au originate from the Be ring and Au TEM grid.6

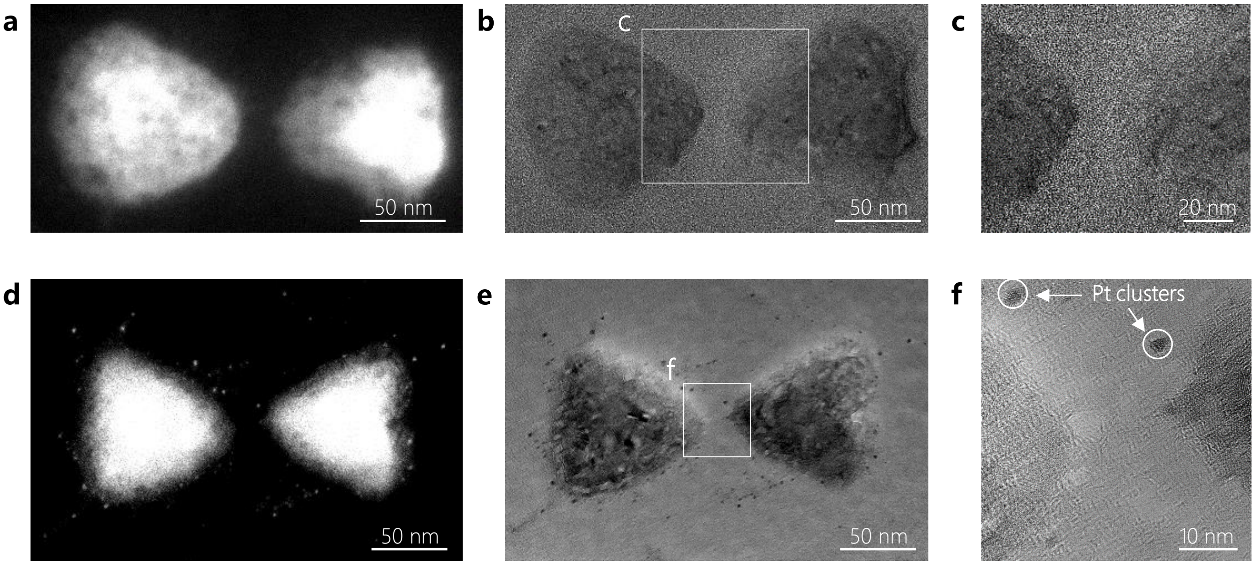

Supplementary Figure S8. (a,b) HAADF-STEM and TEM images of an Al bowtie before thermal annealing. (c) Close up of the area marked on panel b. (d,e) HAADF-STEM and TEM images of an Al bowtie after thermal annealing. (c) Close up of the area marked on panel b.

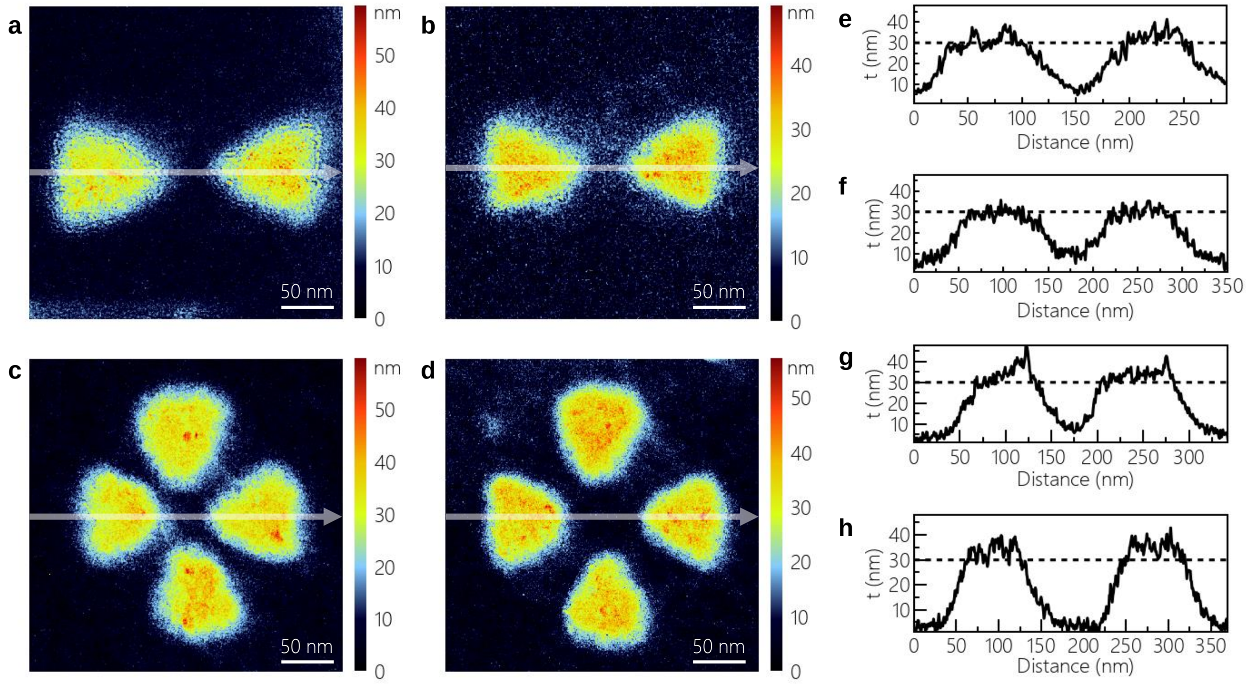

Supplementary Figure S9. (a-d) Thickness maps of four different Al nanocavities on graphene. (e-h) Line profiles taken along the white arrows on the panels a-d, respectively.

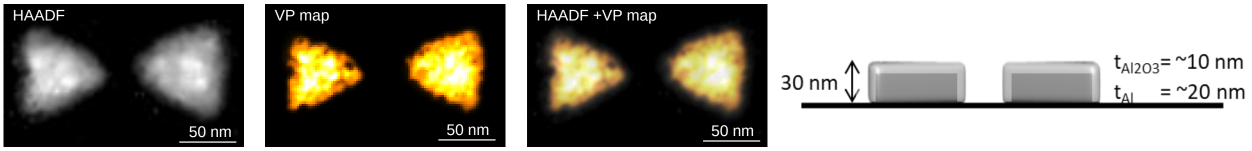

Supplementary Figure S10. HAADF-STEM image, volume plasmon map and superimposed HAADF and volume plasmon map of an Al bowtie. Schematic shows the estimated thickness of the oxide cover on Al. Since volume plasmons exist only in the metallic Al, the spatial difference between the volume plasmon map and HAADF image of nanocavities can provide information about the thickness of amorphous AlO_y layer covering the metallic surface. The superimposed images show that the thickness of oxide layer varies and it is approximately 10 nm.

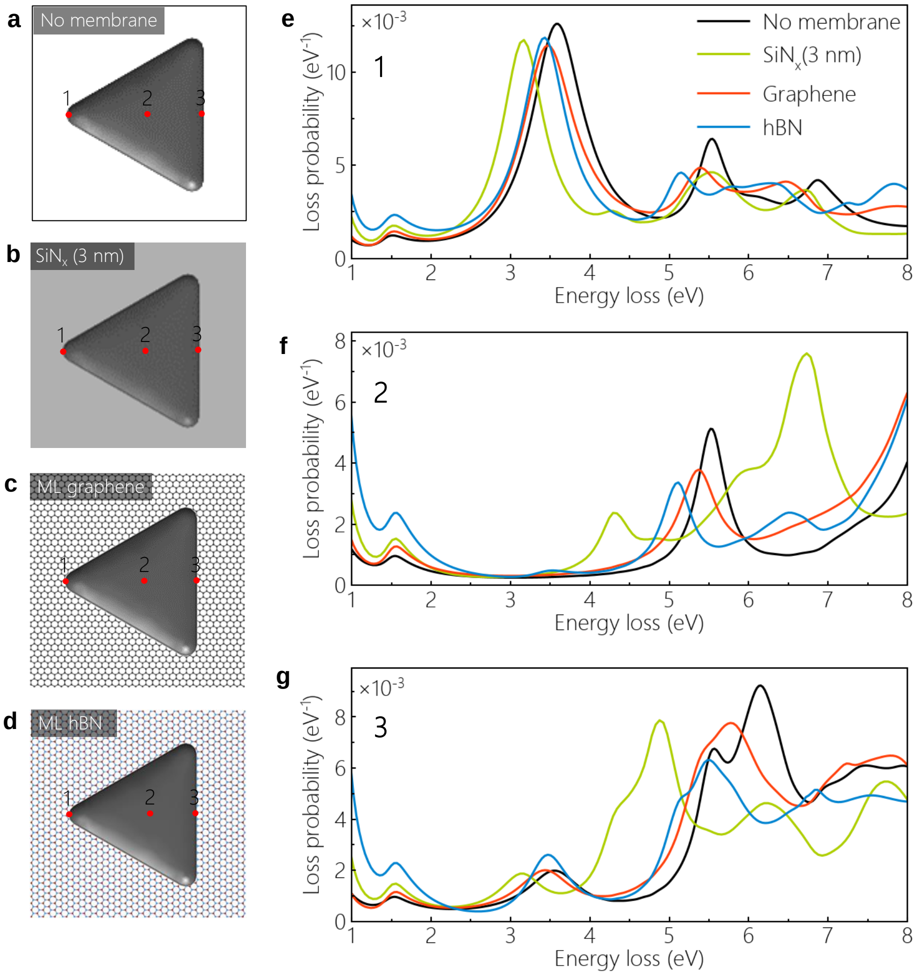

Supplementary Figure S11. (a-d) Simulated EEL spectra of an Al nanoprism in vacuum, on a 3 nm-thick SiN_x, on a ML graphene and on a ML hBN membrane, respectively.

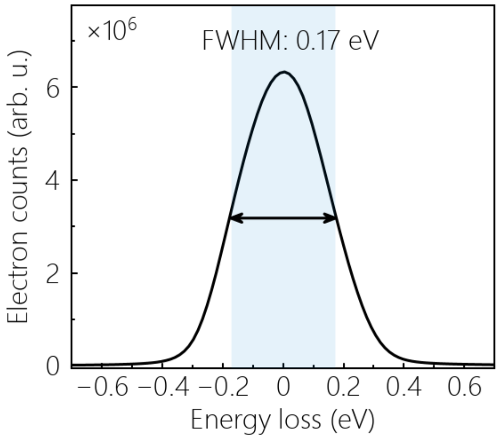

Supplementary Figure S12. ZLP spectra recorded in vacuum.

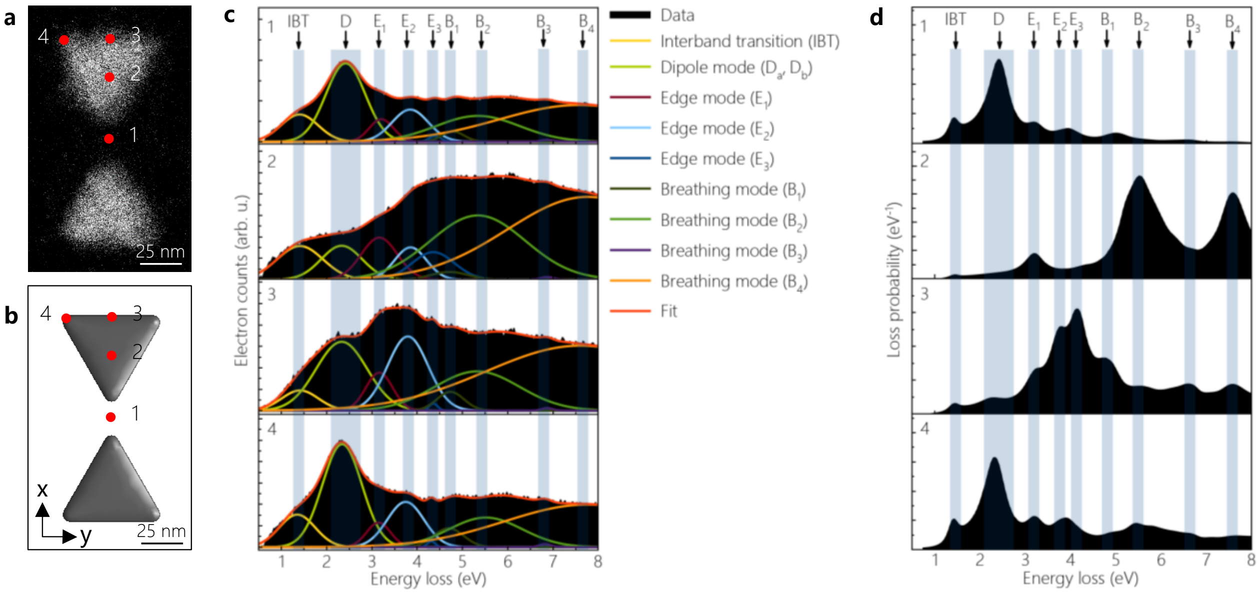

Supplementary Figure S13. (a,b) HAADF-STEM image of an Al bowtie on graphene and its corresponding model used in BEM simulations. (c,d) Experimental and simulated EEL spectra acquired at different positions on the HAADF image and model (see red dots). The background in experimental data is subtracted by power-law fitting.

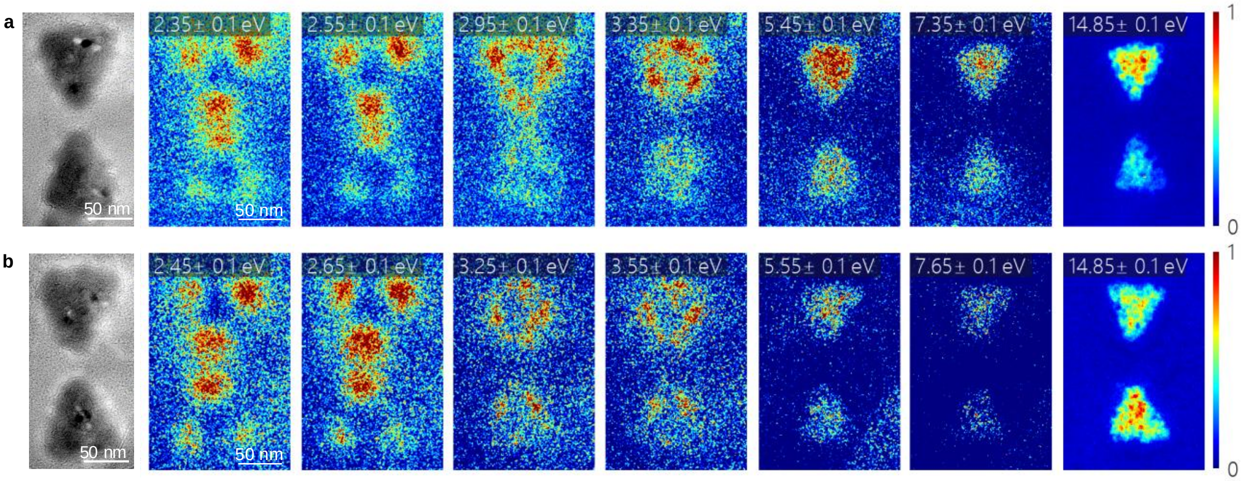

Supplementary Figure S14. (a,b) EFTEM maps acquired at different energies for two Al bowties with the gap sizes of 23 nm and 24 nm, respectively.

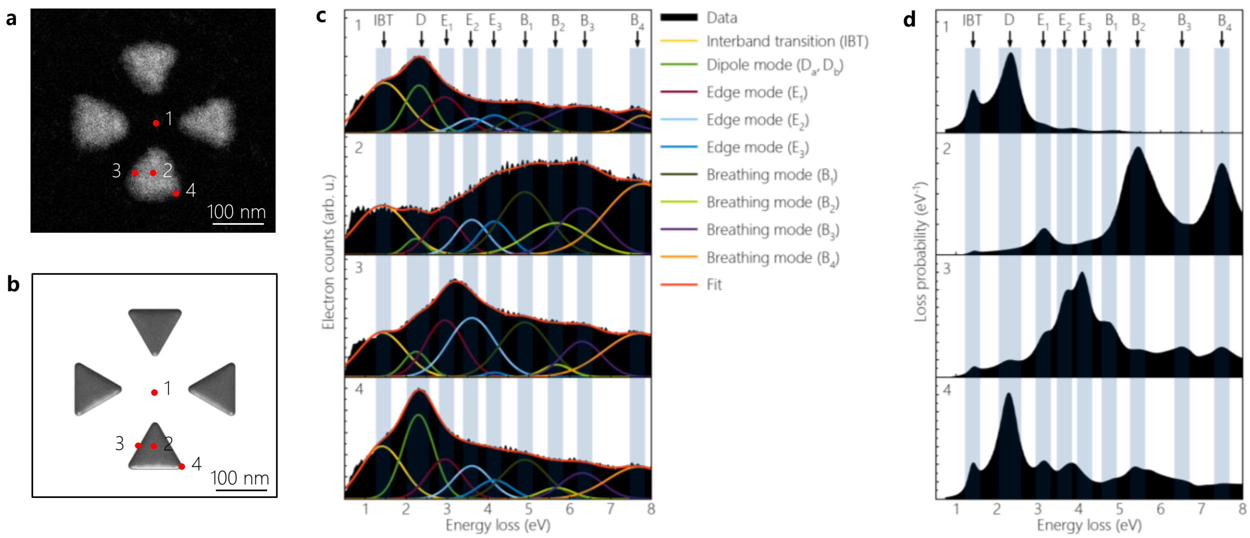

Supplementary Figure S15. (a,b) HAADF-STEM image of an Al tetramer on graphene and its corresponding model used in BEM simulations. (c,d) Experimental and simulated EEL spectra acquired at different positions on the HAADF image and model (see red dots). The background in experimental data is subtracted by power law fitting.

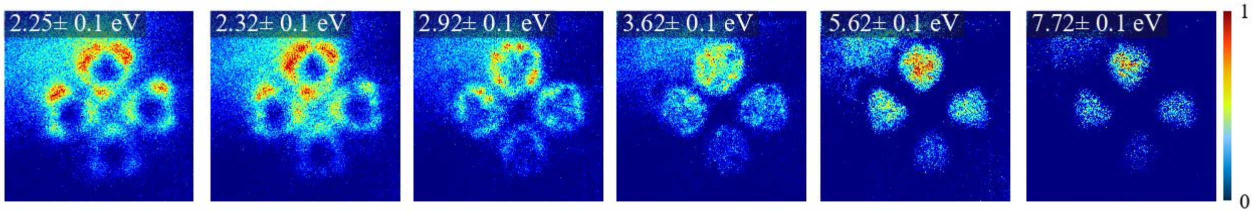

Supplementary Figure S16. EFTEM maps acquired at different energies for the Al tetramer shown in Figure 6a.

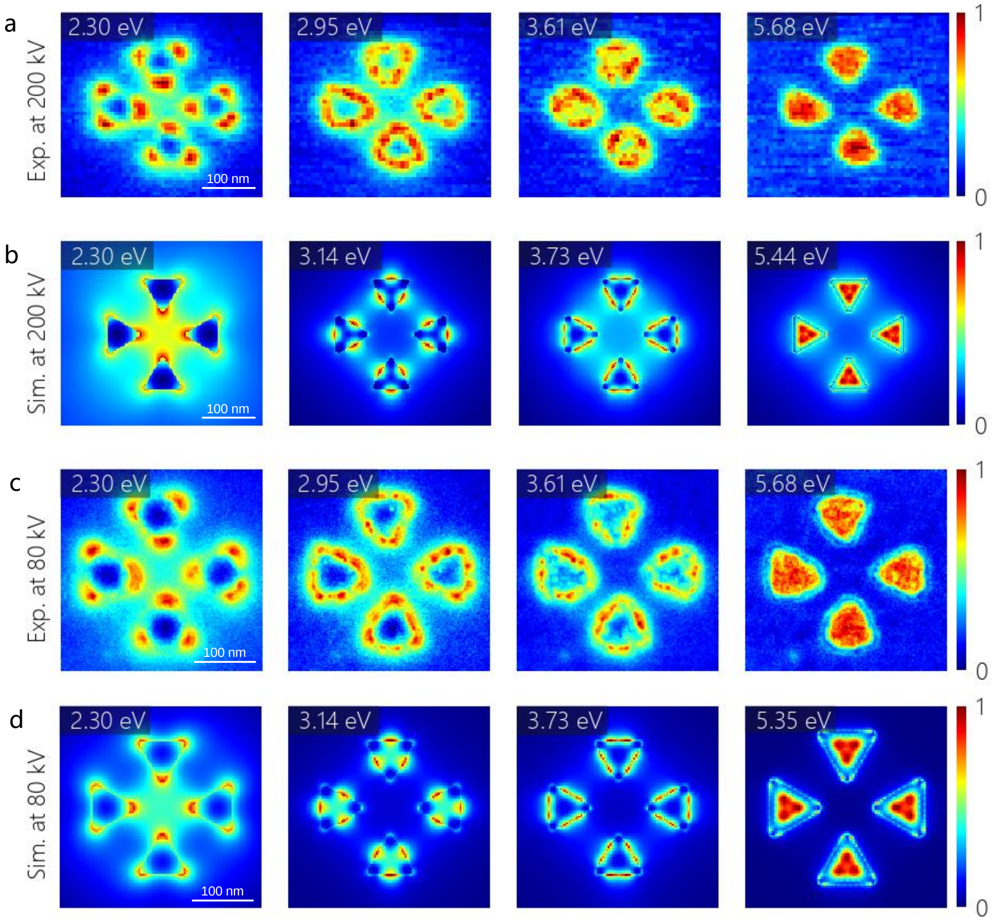

Supplementary Figure S17. Spatially resolved (a,c) experimental and (b,d) simulated EELS maps of an Al tetramer acquired at different energies. The experiments in panels a and c have been performed at 200 kV via a monochromated STEM and 80 kV via a C_s-corrected STEM without a monochromator. Simulations in panels b and d have been performed at 200 kV and 80 kV for the same Al tetramer structure, respectively.

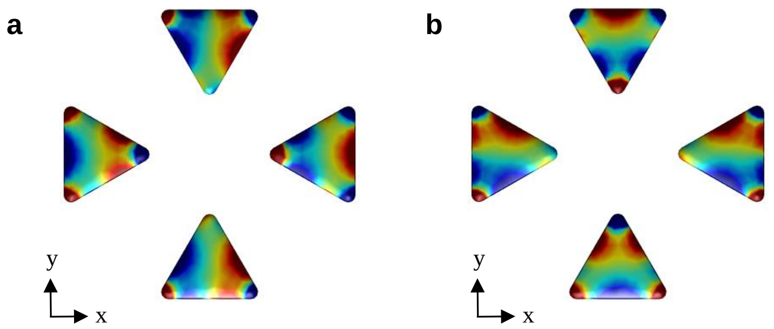

Supplementary Figure S18. (a,b) Simulated surface-charge distributions calculated using an *x*- and *y*-polarized plane wave excitation with the energy of 2.30 eV.

Supplementary Table 1. Experimental and simulated energies of LSPR modes of Al bowtie and tetramer nanocavities shown in Supplementary Figures 13 and 15.

Mode	Bowtie (exp.)	Bowtie (sim.)	Tetramer (exp.)	Tetramer (sim.)
IBT	1.40 ± 0.03 eV	1.41 eV	1.41 ± 0.02 eV	1.41 eV
D_a	2.42 ± 0.01 eV	2.42 eV	2.30 ± 0.36 eV	2.30 eV
D_b	2.34 ± 0.01 eV	2.33 eV	2.32 ± 0.43 eV	2.33 eV
E_1	3.16 ± 0.07 eV	3.2 eV	2.94 ± 0.08 eV	3.14 eV
E_2	3.79 ± 0.12 eV	3.78 eV	3.61 ± 2.16 eV	3.73 eV
E_3	4.31 ± 0.02 eV	4.16 eV	4.16 ± 1.16 eV	4.09 eV
B_1	4.73 ± 0.09 eV	4.73 eV	4.90 ± 1.50 eV	4.64 eV
B_2	5.32 ± 0.18 eV	5.51 eV	5.68 ± 0.04 eV	5.44 eV
B_3	6.86 ± 0.04 eV	6.62 eV	6.30 ± 0.26 eV	6.50 eV
B_4	7.60 ± 0.36 eV	7.58 eV	7.71 ± 0.17 eV	7.49 eV

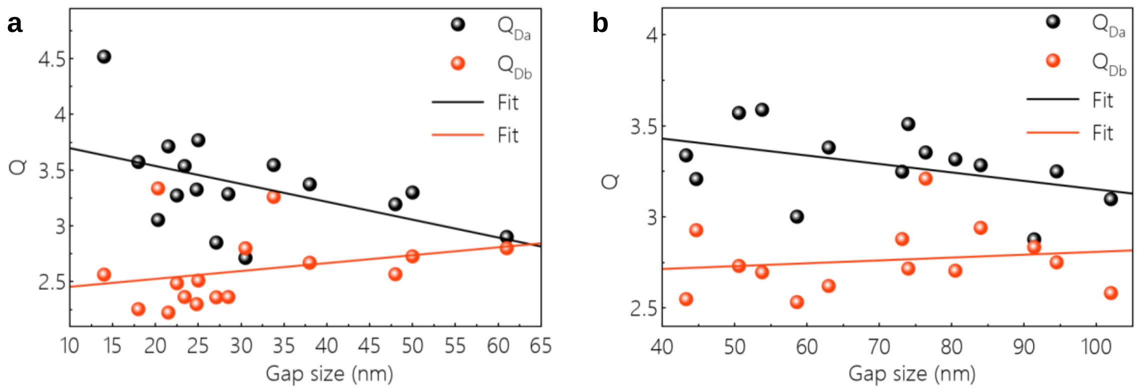

Supplementary Figure S19. (a,b) Quality factors of D_a and D_b modes in bowtie and tetramer nanocavities, respectively.

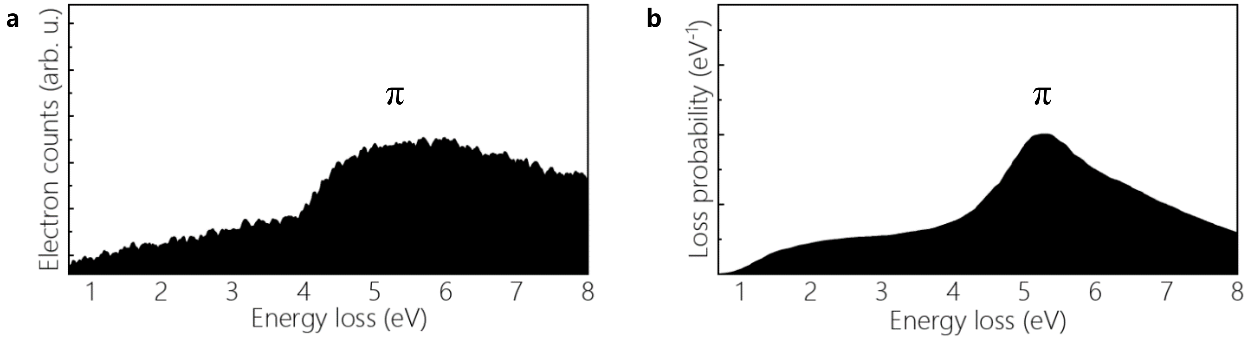

Supplementary Figure S20. (a) EEL spectra recorded on graphene surface far from the bowtie and tetramer nanocavities. (b) Simulated EEL spectra of a ML graphene. The background in experimental data is subtracted by power-law fitting.

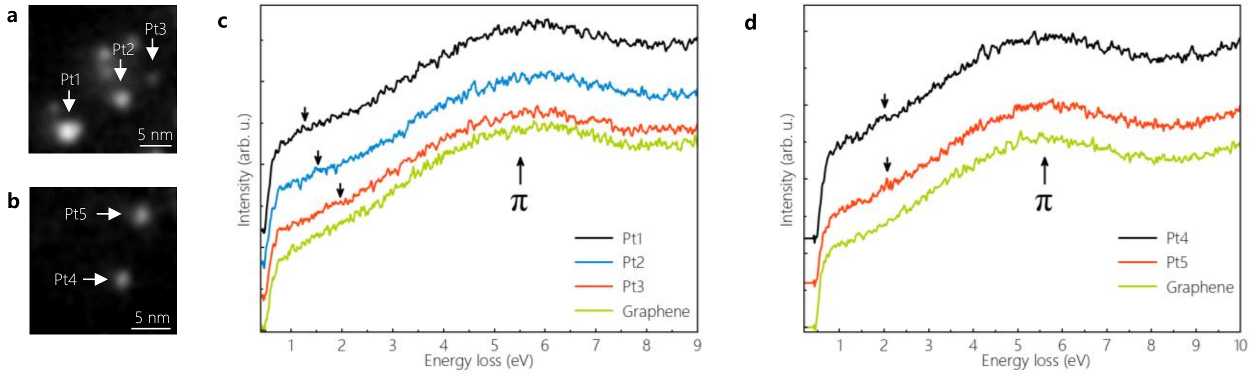

Supplementary Figure S21. (a,b) HAADF-STEM images of Pt NCs with different sizes (the sizes of Pt1, Pt2, Pt3, Pt4 and Pt5 in panels a and b are 3.69, 2.22, 1.33, 1.27 and 1.30 nm, respectively)

on graphene. (c,d) EEL spectra acquired on Pt NCs shown in panels a and b, respectively. The arrows on EEL spectra show the onset of LSPR peaks and π-plasmon of graphene. The background is subtracted by power-law fitting.

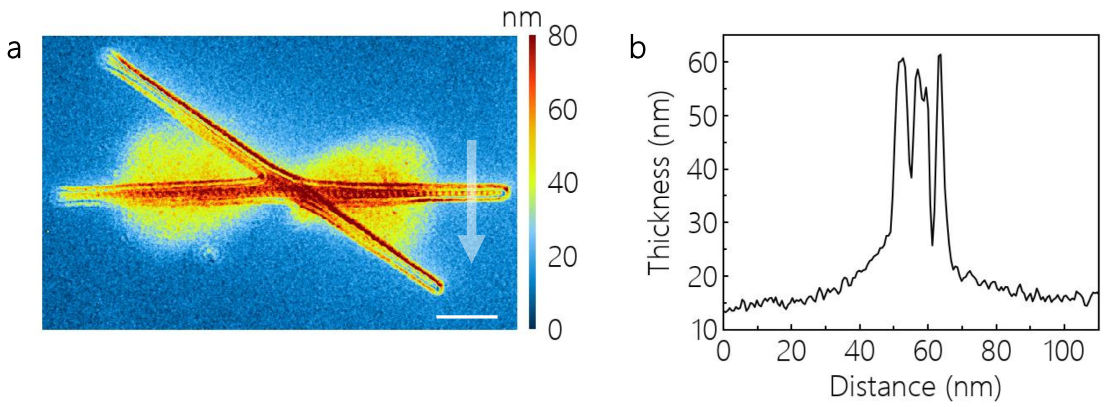

Supplementary Figure S22. (a) Thickness map acquired after EELS line scan for the bowtie shown in Figure 7a. (b) Line profile recorded along the semi-transparent white line on panel a.

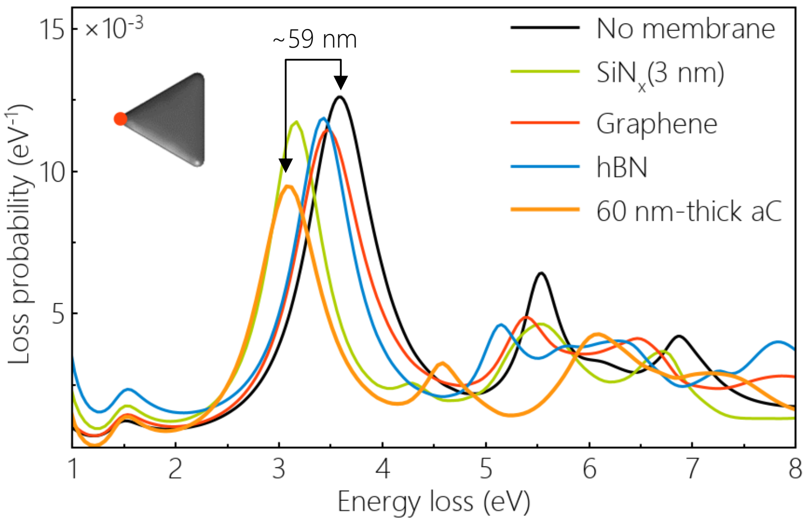

Supplementary Figure S23. Simulated EEL spectra of an Al nanoprism in a vacuum, on a 3 nm-thick SiN_x, on a monolayer graphene, on a monolayer hBN and a 60 nm-thick amorphous carbon layer, respectively. The EEL spectra were obtained from the edge of an Al nanoprism via an electron beam excitation (see the red dot on the nanoprism).

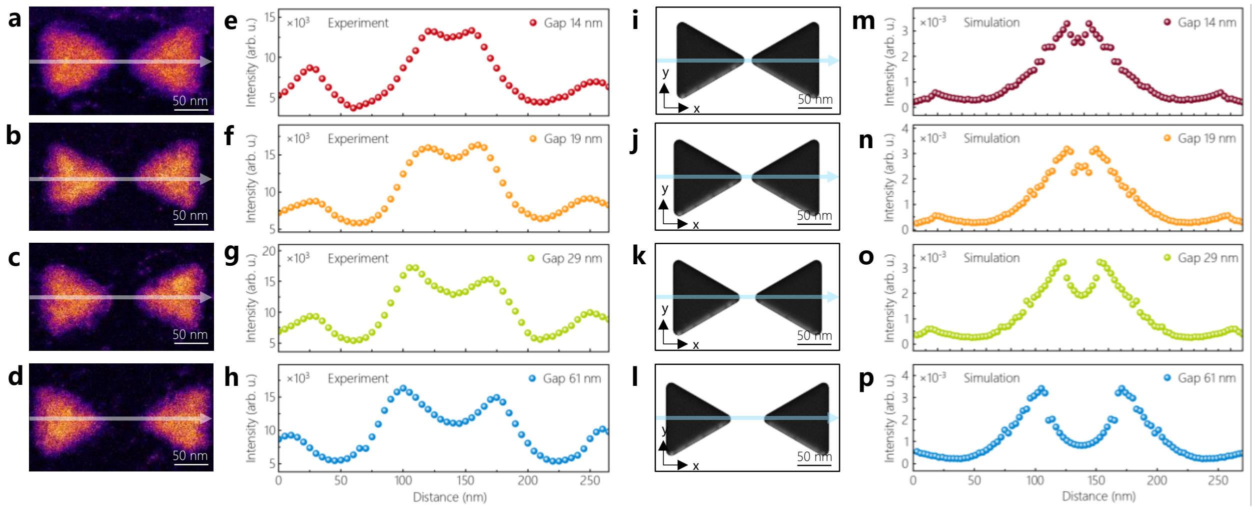

Supplementary Figure S24. (a-d) HAADF-STEM images of Al bowties with different gap sizes on graphene. There is no Pt NC trapped at the hotspots. (e-h) Intensities of dipolar mode excited along the blue transparent lines on the panels a-d, respectively. (i-l) Models corresponding to the experimentally observed structures. (m-p) Intensities of dipolar mode excited along the blue transparent lines on the panels i-l, respectively. Intensity is averaged over an area of 0.2 eV on dipolar modes in experimental and simulated data.

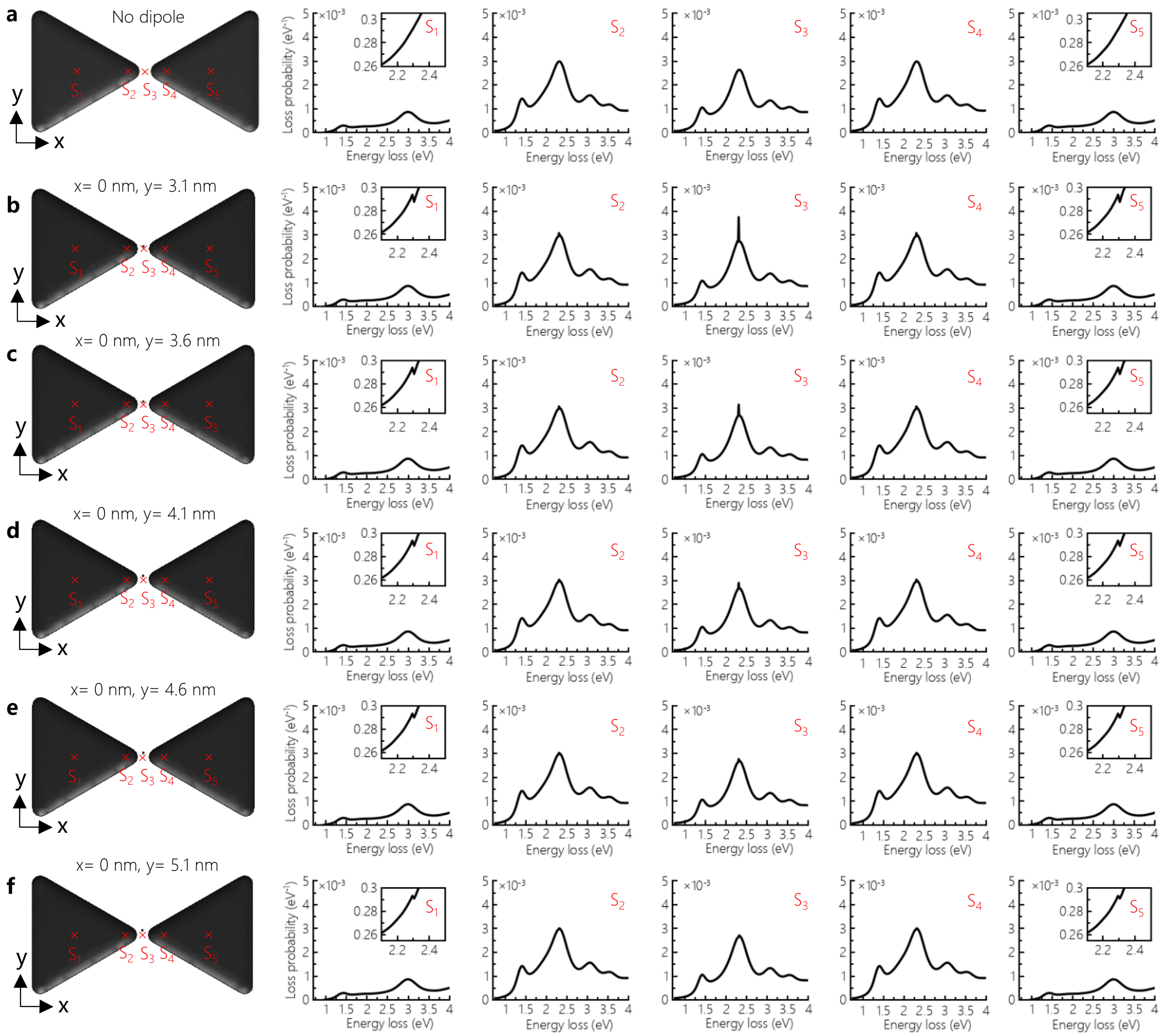

Supplementary Figure S25. (a) Simulated EEL spectra calculated at different positions on an Al bowtie without a dipole at the hotspot. (b,f) Simulated EEL spectra calculated at different positions on an Al bowtie with a dipole trapped at the plasmonic hotspot. The position of the dipole is changed in *y*-direction (*x* = 0 for all) in each panel b-f. The energies of both D_a mode excited at the center (0,0) of nanocavity and dipolar mode of the dipole trapped are 2.32 eV in all configurations.

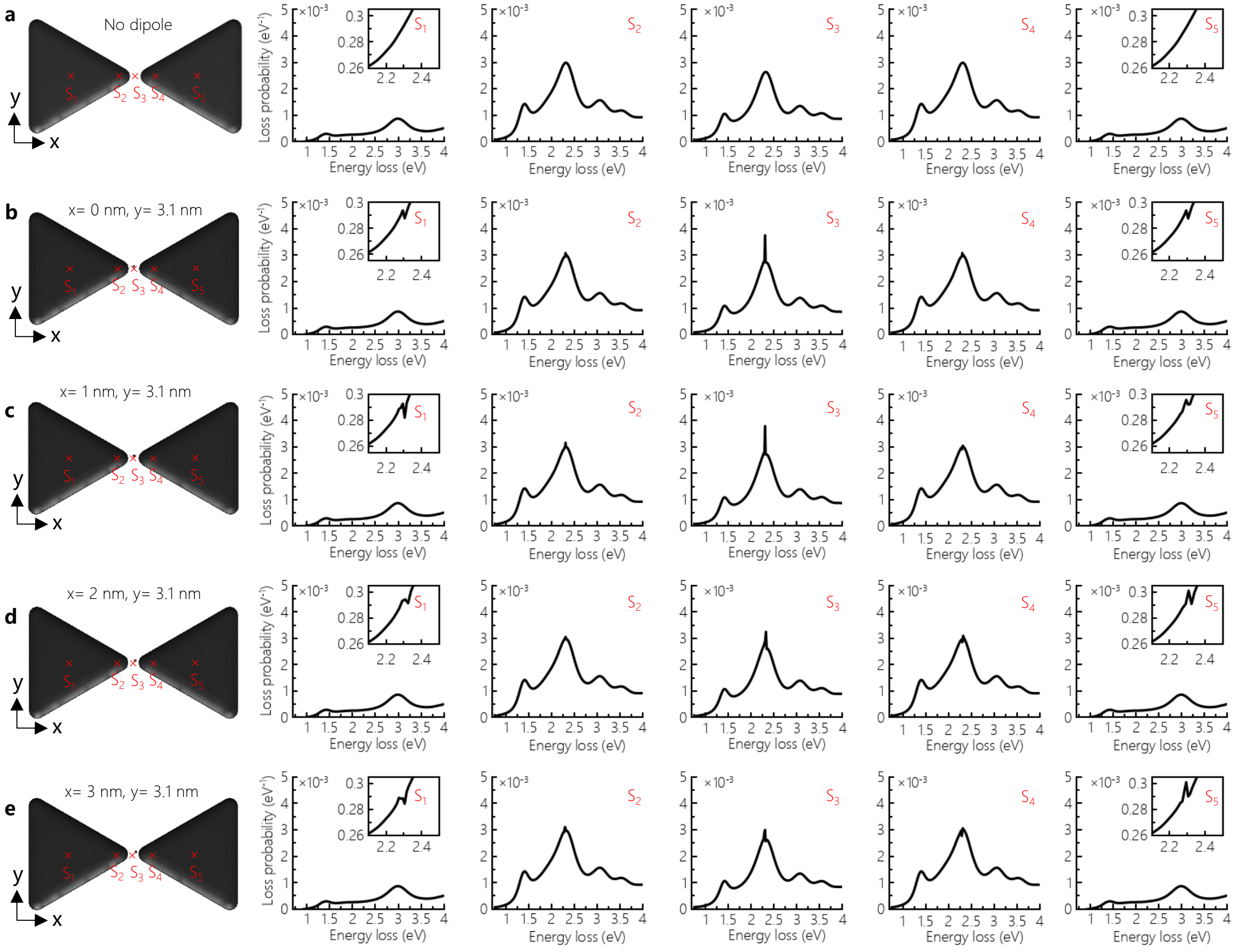

Supplementary Figure S26. (a) Simulated EEL spectra calculated at different positions on the Al bowtie without a dipole at the hotspot. (b,e) Simulated EEL spectra calculated at different positions on the Al bowtie with a dipole at the plasmonic hotspot. The position of the dipole is changed in *x*-direction (y = 3.1 nm for all) in each panel b-e. The energies of both D_a mode excited at the center (0,0) of nanocavity and dipolar mode of the dipole trapped are 2.32 eV in all configurations.

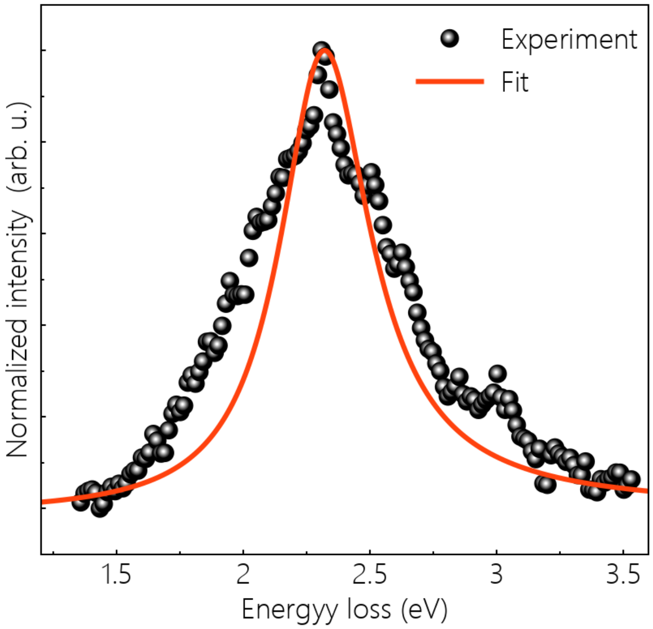

Supplementary Figure S27. EEL spectra fitted to the coupled oscillator model. The following parameters are used in fitting: ω_{cp}= 2.32 eV, ω_{pp}= 2.32 eV, g= 10 meV, δ= 80 meV, γ_{cp} = 429 meV and γ_{pp}= 5.8 meV.

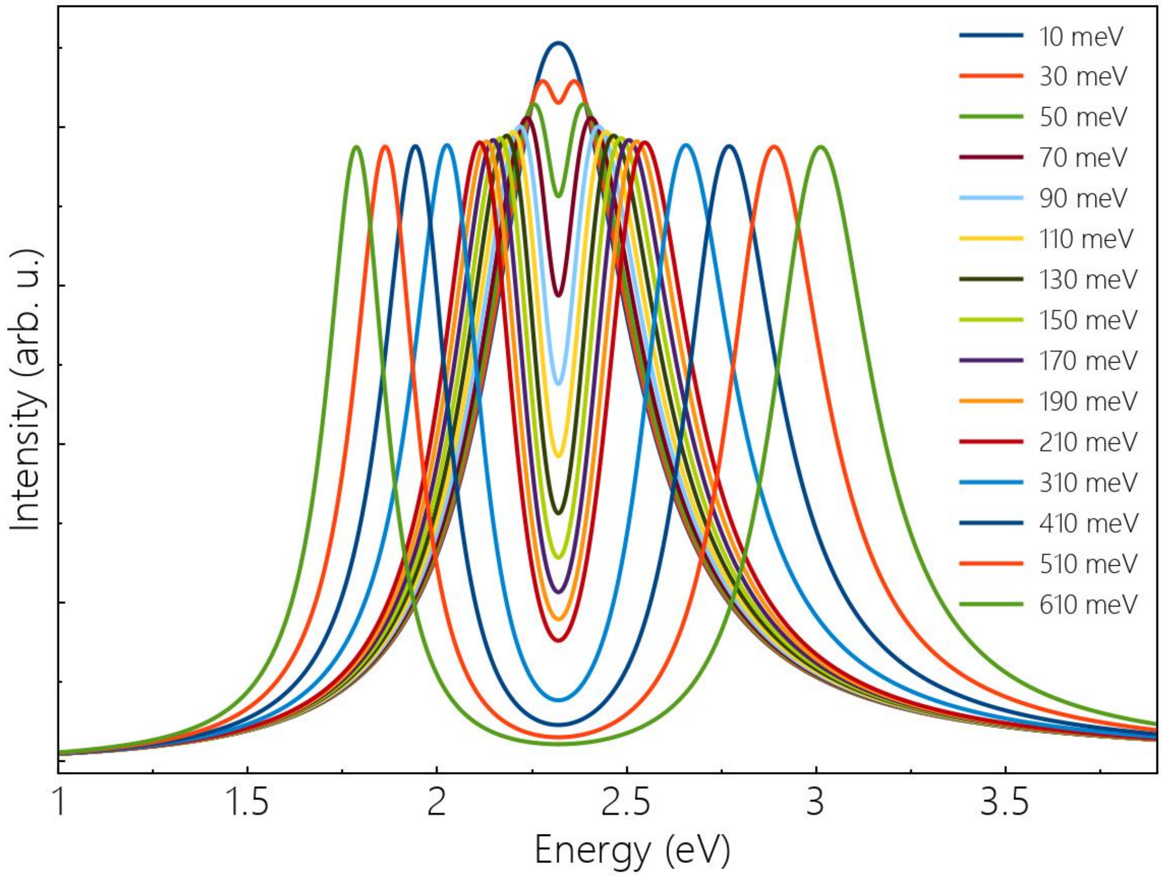

Supplementary Figure S28. Simulated EEL spectra based on the coupled oscillator model. The coupling strength g is varied from 10 meV to 610 meV. Here, ω_{cp}= 2.32 eV, ω_{pp}= 2.32 eV, δ= 80 meV, γ_{cp} = 429 meV and γ_{pp}= 5.8 meV.

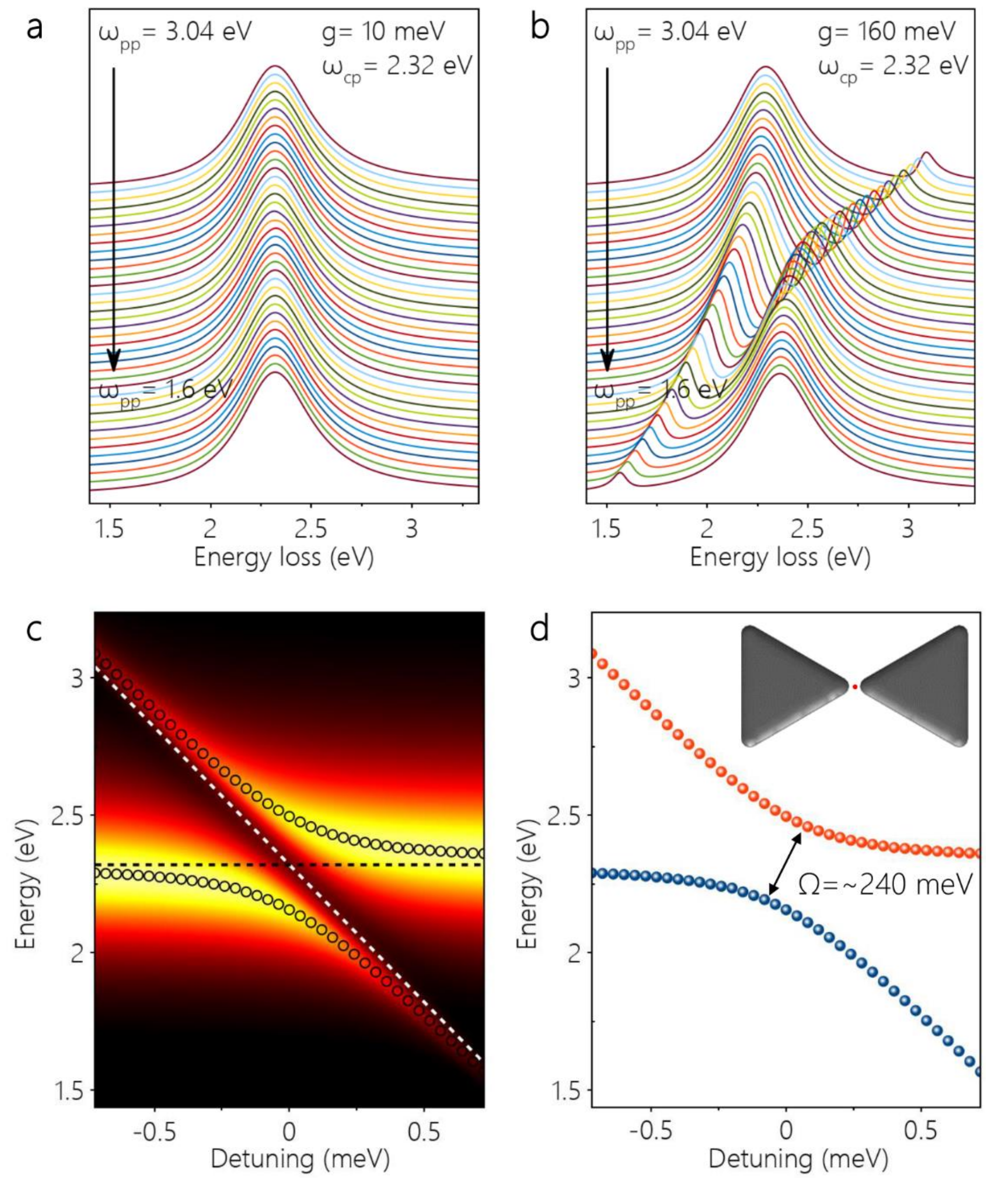

Supplementary Figure S29. (a,b) The simulated EEL spectra obtained from a dipole trapped within the hotspot of a bowtie for g= 10 meV and g= 160 meV. Here, the dipole energy of the Pt NC is varied by changing the dielectric constant of the cover layer surrounding it, while ω_{cp}= 2.32 eV. (c) Contour plot of the EEL spectra obtained for g = 160 meV as a function of detuning ($\omega_{cp} - \omega_{pp}$). The detuning is obtained by changing the resonant energy of the Pt NC within the hotspot. The white and black dashed lines show the resonant energies of the dipolar mode excited at the center of the hotspot and the dipolar mode in the Pt NC. The circles demonstrate the energies of upper and lower braches as a function of detuning. (d) The energies of upper and lower braches as a function of detuning. The inset shows the dipole (red dot) located within the nanocavity.

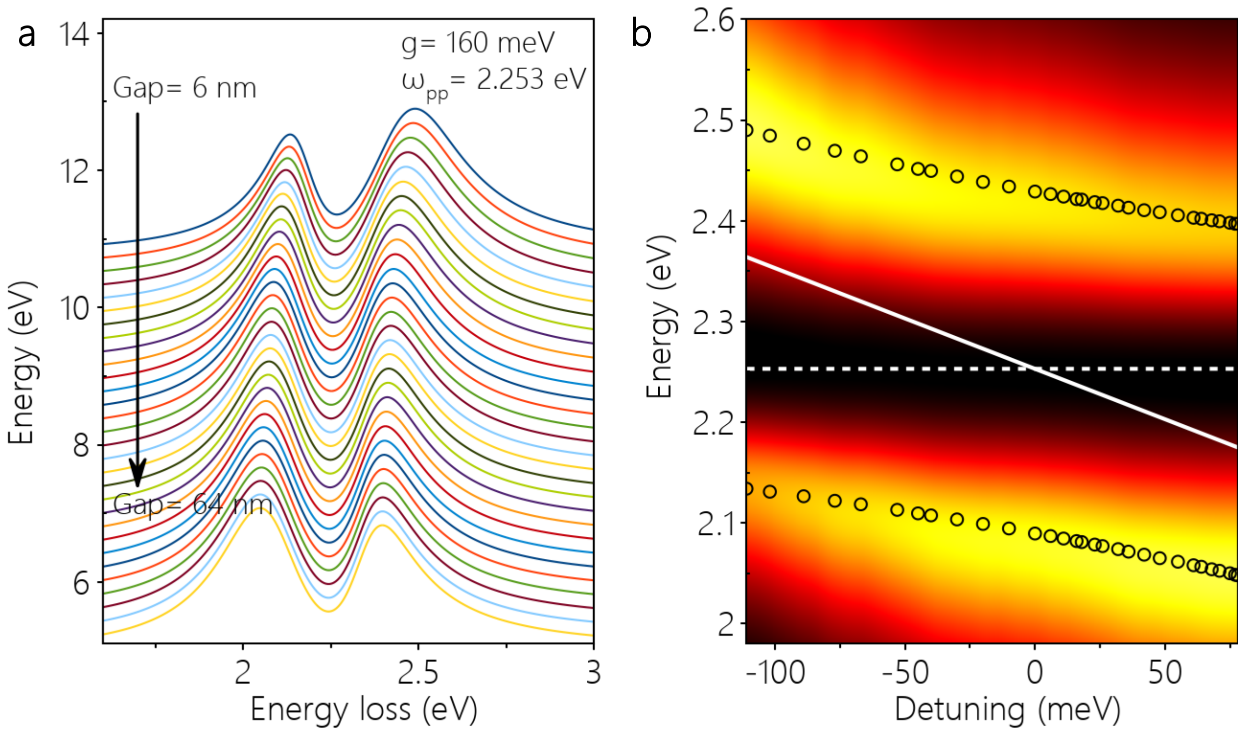

Supplementary Figure S30. (a) The simulated EEL spectra obtained from a dipole placed at the center of the hotspot in bowties with different gap sizes for g= 160 meV. Here, the dipole energy of the bowties is varied by changing their gap sizes while ω_{pp}= 2.253 eV. (b) Contour plot of the EEL spectra obtained for g = 160 meV as a function of detuning $(\omega_{cp} - \omega_{pp})$. The detuning is obtained by changing the gap size of the bowties. The solid and dashed white lines show the resonant energies of the dipolar mode excited at the center of nanocavity and the dipolar mode excited at the Pt NC. The circles demonstrate the energies of upper and lower braches as a function of detuning.

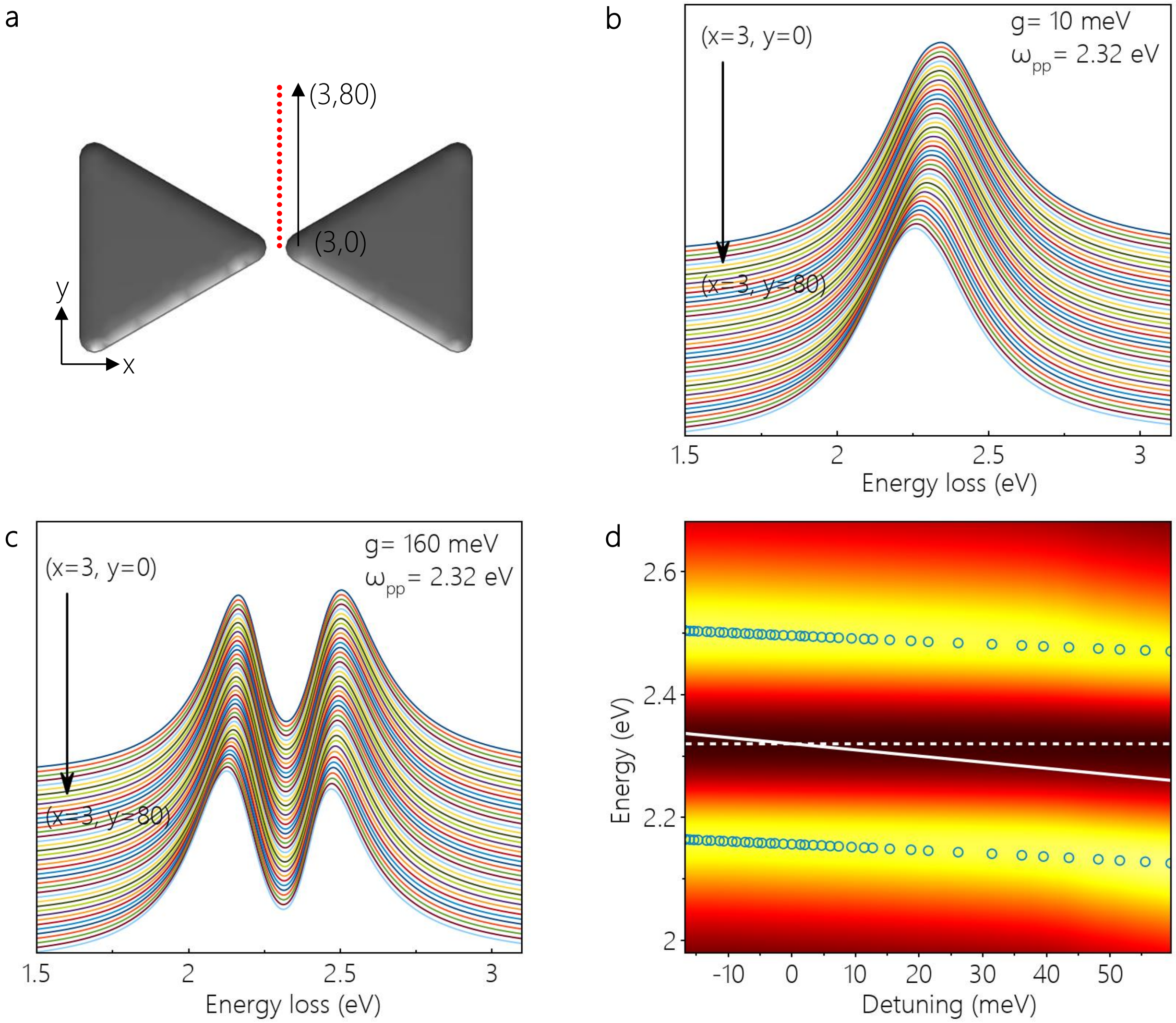

Supplementary Figure S31. (a) A model showing the bowtie with a dipole located at different positions (from y= 0 nm to y= 80 nm) along the y-axis, while its position on x-axis is stationary (x= 3 nm). (b,c) The simulated EEL spectra obtained from different positions, where the dipole is located within the hotspot while g= 10 meV and g= 160 meV. (d) Contour plot of the EEL spectra obtained for g = 160 meV as a function of detuning ($\omega_{pp} - \omega_{cp}$). The white dashed and solid lines show the resonant energies of the dipolar mode excited at the Pt NC and the dipolar mode excited at the center of the hotspot. The detuning is obtained by changing the location of the dipole within the hotspot along the y-axis. The circles demonstrate the energies of upper and lower branched as a function of detuning.

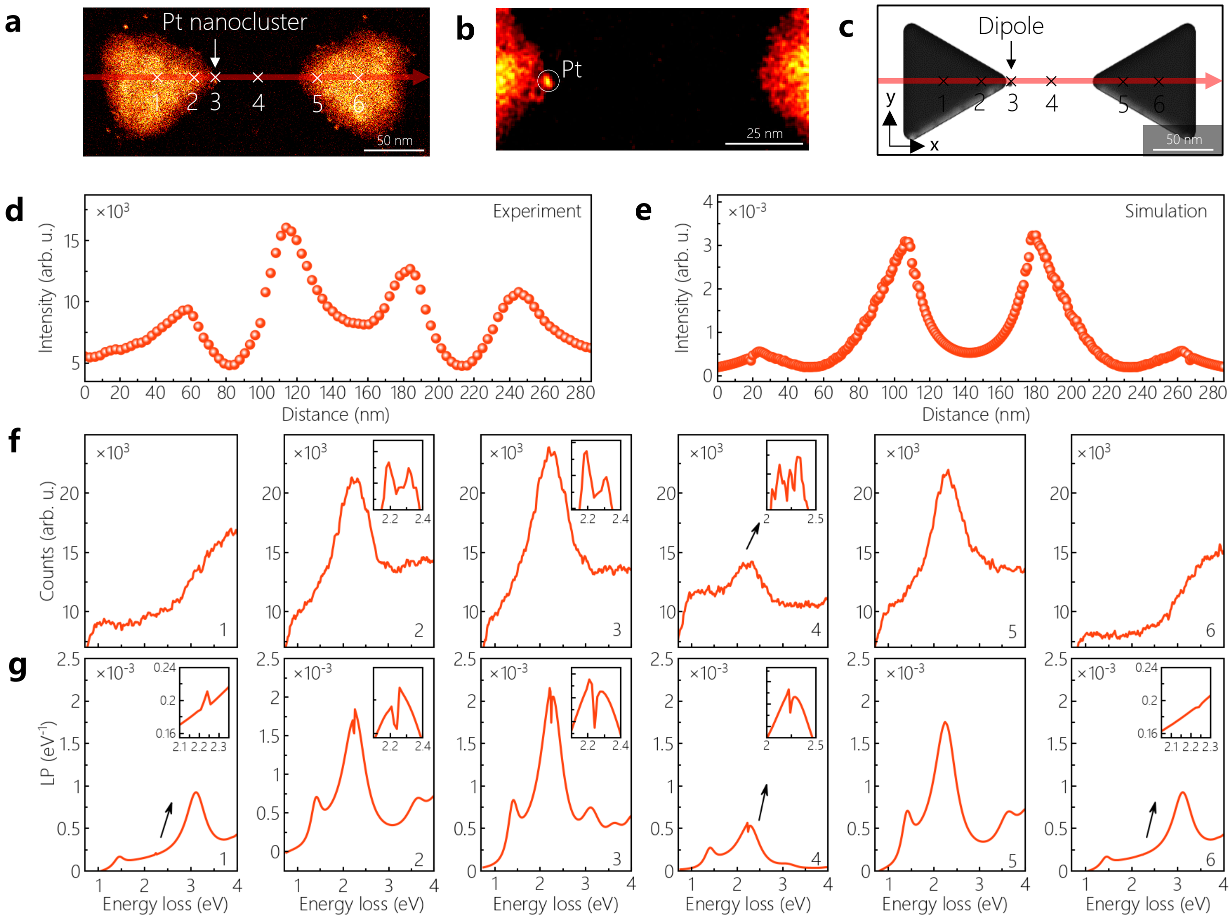

Supplementary Figure S32. (a,b) Overview and close-up HAADF-STEM images of an Al nanocavity with a Pt NC (~ 1.78 nm) trapped within the plasmonic hotspot. The close-up HAADF image is Gaussian blurred with a 1 px. (b) A model corresponding to the experimentally observed structure. The gap size is 68 nm in both experiments and simulations. (d,e) Experimental and simulated intensities of dipolar mode excited along the red transparent lines on the panels a and c. Intensity is averaged over an area of 0.1 eV on dipolar modes in experimental and simulated data. (f,g) Experimental and simulated EEL spectra obtained on the marked positions on panels a and c. The energies of D_a mode excited at the center of the hotspot (position 4 in panel a) and the dipole trapped are set to 2.26 eV and 2.252 eV in the BEM simulations. The background in the experimental data is subtracted by power-law fitting.

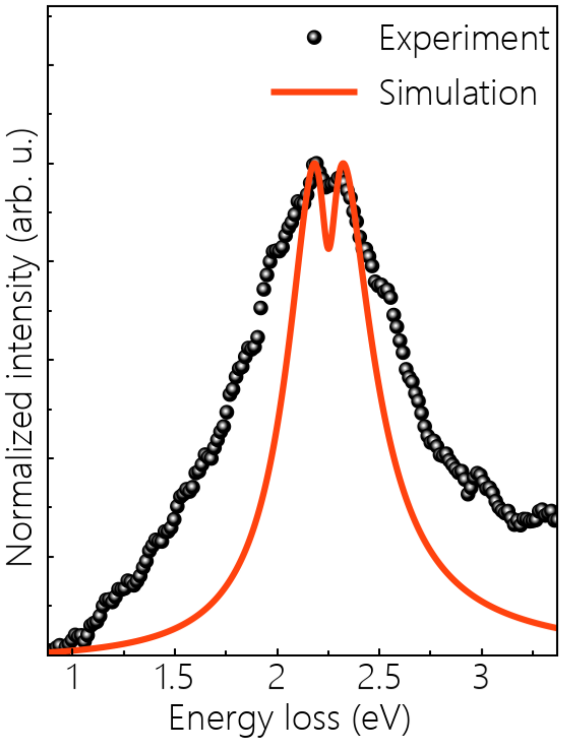

Supplementary Figure S33. (a-c) EEL spectra fitted to the coupled oscillator model. The EEL spectra in panels a-c are taken from position 3 in Supplementary Figure 32f. The following parameters are used in fitting: ω_{cp}= 2.252 eV, ω_{pp}= 2.252 eV, g= 55 meV, δ= 80 meV, γ_{cp} = 429 meV and γ_{pp}= 5.8 meV.

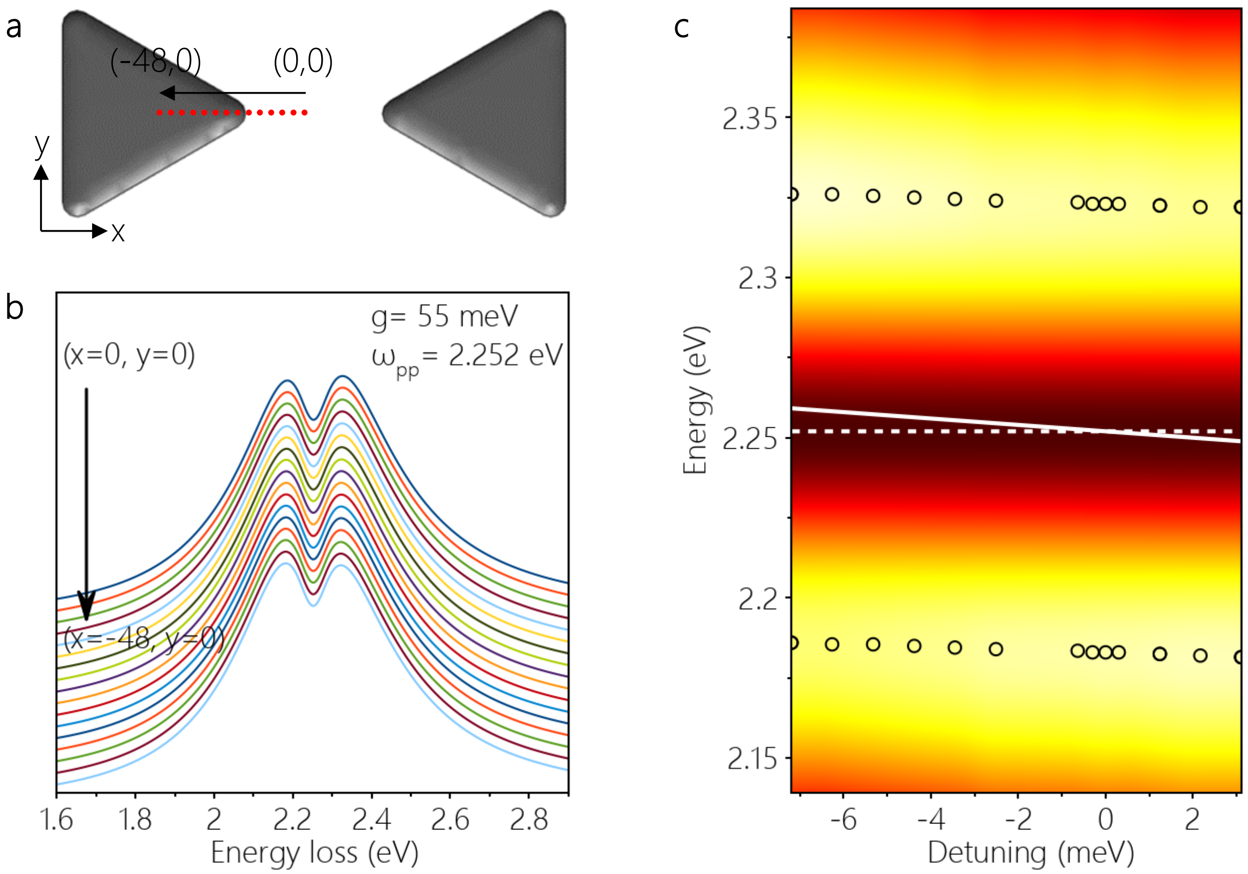

Supplementary Figure S34. (a) A model showing the bowtie with a dipole located at different positions (from x= 0 nm to x= 80 nm) along the x-axis, while its position on the y-axis is stationary (y= 0 nm). (b) The EEL spectra obtained from different positions, where the dipole is located within the hotspot. (c) Contour plot of EEL spectra obtained for g = 55 meV as a function of detuning ($\omega_{pp} - \omega_{cp}$). The detuning is obtained by changing the location of the dipole within the hotspot along the x-axis. The white dashed and solid lines show the resonant energies of the dipolar mode excited at the Pt NC and the dipolar mode excited at the center of the hotspot. The circles demonstrate the energies of upper and lower branches as a function of detuning. Both panels b and c are obtained using the following parameters: g = 55 meV, ω_{pp}= 2.252 eV.
